# Groundwater delivery of neonicotinoids to streams

**DOI:** 10.1002/jeq2.70234

**Published:** 2026-08-16

**Authors:** William M. DeVita, Paul M. McGinley

**Affiliations:** ^1^ Center for Watershed Science and Education, College of Natural Resources University of Wisconsin‐Stevens Point Wisconsin Stevens Point USA

## Abstract

Neonicotinoids are mobile and persistent insecticides whose presence in streams poses a threat to stream invertebrates. This study sought to understand how stream baseflow concentrations of three common neonicotinoids relate to their delivery in groundwater from agricultural land. In a study area with large areas of irrigated potato (*Solanum tuberosum* L.) and vegetable crops, some grain and dairy agriculture, and small areas of suburban development, neonicotinoid concentrations in streams exceeded the aquatic life benchmarks for imidacloprid and clothianidin at the sites with higher percentages of potato and vegetable crops in their groundwater contributing area. The stream concentrations are similar to field‐edge groundwater concentrations after adjusting for the density of agricultural land cover within a groundwater travel‐time corresponding to their usage period and assuming the neonicotinoids persist or degrade slowly in the aquifer. If neonicotinoids do not degrade in the aquifer, the stream concentrations can be explained by a mean loss to groundwater from the land in potato and vegetable agriculture of 0.16 (range 0.002–0.3) g ha^−1^ year^−1^ for imidacloprid, 1.6 (range 0.02–4.1) g ha^−1^ year^−1^ for thiamethoxam, and 0.5 (range 0.05–1.0) g ha^−1^ year^−1^ for clothianidin. Given that only approximately one‐third to two‐thirds of the water entering the streams was young enough to contain these neonicotinoids, if neonicotinoid loss to groundwater stays the same and they persist in the aquifer, their concentrations will continue to increase as those streams come to steady‐state with applied neonicotinoids.

## Plain Language Summary

Neonicotinoids are insecticides used to control pests in agriculture and landscaping but they also impact other insects. This research explored how neonicotinoids might move from the land where they are applied into groundwater and then travel in groundwater to streams where they could affect insects in the streams. We measured neonicotinoid concentrations in streams within a sandy agriculture area and compared those concentrations to the type and intensity of crops grown where the groundwater that moves to these streams originates. Our results show that the areas with higher intensity of irrigated vegetable and potato crops had higher concentrations of neonicotinoids, consistent with the groundwater delivery of land‐applied neonicotinoids to the streams.

**Abbreviations**
ALB: aquatic life benchmark
EPA: US Environmental Protection Agency
LC/MS/MS: liquid chromatography/tandem mass spectrometry
MODFLOW: Modular Groundwater Flow Model
MODPATH: particle‐tracking model for MODFLOW
POCIS: polar organic chemical integrative sampler
WCS: Wisconsin Central Sands

## 1 INTRODUCTION

Neonicotinoids are an important group of insecticides with specific toxicity to insects and other arthropods. They have only been used since the 1990s, but within 25 years, they constituted more than 25% of global insecticide market (Bass et al., 2015). They are applied through foliar and furrow applications and increasingly as coatings on seeds. Neonicotinoids are water‐soluble systemic insecticides translocated through the plant during growth. Their mode of action can lead to impacts on nontarget organisms such as pollinators and other insects in agricultural fields and adjacent areas (Montiel‐León et al., 2019; Sanchez‐Bayo & Goka, 2014). Neonicotinoids can persist in the environment and move from areas of application into surface water (Pietrzak et al., 2020). Neonicotinoids move to surface waters in surface runoff, tile drainage, dust, and drift during planting and foliar application and by percolation through the soil and eventual transport in groundwater (Alford & Krupke, 2019; Bonmatin et al., 2015; Goedjen et al., 2024; Huseth & Groves, 2014; Main et al., 2014; Schaafsma et al., 2019; Thompson et al., 2021; Wettstein et al., 2016). In some watersheds, stream concentrations show higher neonicotinoid concentrations in spring and summer, reflecting movement in surface runoff (Berens et al., 2021; Hladik et al., 2014). Similarly, neonicotinoids in soil percolate, tile drains, and field‐edge groundwater monitoring wells show seasonal patterns consistent with higher concentrations moving through soil to groundwater after planting and harvest (Alford & Krupke, 2019; D. Browne et al., 2021; Huseth & Groves, 2014). At the watershed scale, year‐round detections of neonicotinoids in streams have been attributed to their delivery to the stream in groundwater from multiple sources and across long distances, leading to more uniform year‐round concentrations (Hladik et al., 2018; Miller et al., 2025). Delivery to streams in groundwater can chronically expose aquatic organisms to neonicotinoids. Current US Environmental Protection Agency (EPA) chronic and acute aquatic life benchmarks (ALBs) for aquatic invertebrates are shown in Table 1 (United States Environmental Protection Agency, 2019). Miller et al. (2025) found more than 40% of streams monitored throughout the United States from 2017 to 2022 had imidacloprid concentrations exceeding the chronic ALB. Concentrations were elevated year‐round and increasing in many of those watersheds, consistent with their delivery to the streams from groundwater. Developing a better understanding of groundwater delivery of neonicotinoids to streams is important as our understanding of the toxicity continues to suggest that these compounds can pose significant hazards to aquatic ecosystems. Both Morrissey et al. (2015) and Tennekes (2010) suggest ecological damage might be expected to populations of aquatic invertebrates at concentrations lower than the EPA ALB with Morrissey et al. proposing ALBs that are the sum of all neonicotinoids. They proposed an acute total neonicotinoid ALB of 200 ng L^−1^ and a chronic total neonicotinoid ALB of 35 ng L^−1^.

**TABLE 1:** US EPA Aquatic Life Benchmarks—Invertebrates (United States Environmental Protection Agency, 2019).

Neonicotinoid	Acute^a^ (ng L^−1^)	Chronic^b^ (ng L^−1^)
Clothianidin	11,000	50
Imidacloprid	385	10
Thiamethoxam	17,500	740

Abbreviation: EPA, Environmental Protection Agency.

^a^ Acute invertebrate toxicity value is usually the concentration resulting in an effect or mortality in 50% of the test organisms.

^b^ Chronic invertebrate toxicity value is usually the lowest no observable adverse effect concentration from life‐cycle tests with invertebrates.

The purpose of this study was to develop a watershed‐scale understanding of the significance of groundwater conveyance of neonicotinoids to streams. We examined streams within the Wisconsin Central Sands (WCS), an area that includes irrigated potato and vegetable agriculture, some dairy and grain production, and small areas of suburban development. The WCS has a groundwater‐dominated stream network that drains an aquifer of sandy glacial outwash and moraine deposits (Weeks & Stangland, 1971). Imidacloprid has been used in the WCS since the mid‐1990s, thiamethoxam has been used since approximately 2000, and clothianidin since approximately 2005 (Wieben, 2021). Previous research has shown streamflow in the WCS includes groundwater that originated as precipitation more than 50 years earlier (B. A. Browne & Guldan, 2005; Tesoriero et al., 2013; Werner et al., 2012). Neonicotinoids have been detected in the groundwater adjacent to agricultural fields and in the streams of the WCS (Bradford et al., 2018; Senger et al., 2019). We hypothesize that the neonicotinoids in groundwater originating within agricultural fields in the WCS lead to their detection in streams and may pose a long‐term threat to aquatic ecosystem health. Our objectives in this study were to (1) measure neonicotinoid concentrations at multiple stream locations in the WCS using both discrete and time‐integrated methods and compare with ALB concentrations, (2) evaluate if the stream concentrations can be explained by landcover in the groundwater contributing areas, and (3) explore how the stream concentrations may change in the future.

## 2 MATERIALS AND METHODS

### 2.1 Sampling locations

**FIGURE 1:**
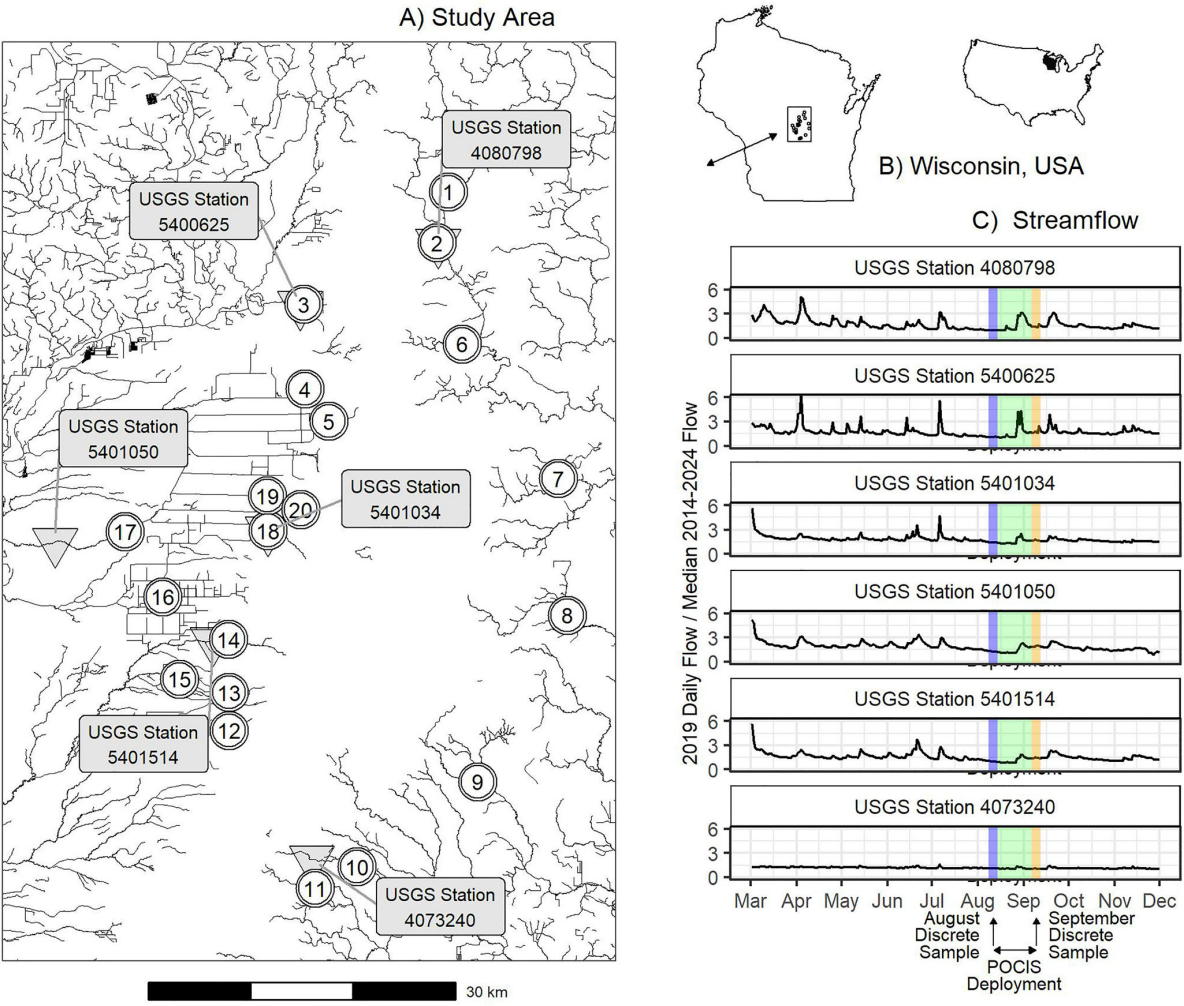
Study area (A) showing the location of sampling sites with site numbers (circles) and the US Geological Survey streamgages (triangles), location within central Wisconsin (B), and streamflow during the sampling year and within the sampling period relative to the median historical flow (C). POCIS, polar organic chemical integrative sampler.

The 20 sampled locations were on streams in the WCS (Figure 1). These are baseflow‐dominated streams where the long‐term average fraction of streamflow that is groundwater is estimated at 84% to more than 90% (Tesoriero et al., 2013; Weeks et al., 1965). The WCS has highly transmissive soils that lead to little overland flow following precipitation events and there are few subsurface tile drains. One of the sites (Site 8) has a municipal wastewater treatment discharging approximately 100,000 gallons day^−1^ upstream of the sampling location. All the other wastewater generated upstream of the sampling sites is discharged to groundwater either through municipal seepage cells after treatment (three communities) or household septic systems. Streamflow during the project period was close to the historical median flow and within a factor of four following a mid‐study rainfall (Figure 1). These hydrologic conditions provide an opportunity to evaluate groundwater delivery of agricultural neonicotinoids to the streams. The strong connection between groundwater and streams in the WCS has been characterized through groundwater flow modeling, chemical age dating, and chemical source tracking (Bradbury et al., 2017; Kraft et al., 2012; Tesoriero et al., 2007).

### 2.2 Neonicotinoid sampling and analysis

At each site, samples were collected using a polar organic chemical integrative sampler (POCIS) deployed for approximately 30 days between late August and late September and two discrete samples, one collected at the beginning and one at the end of the deployment period. Discrete samples were collected in 1‐L amber borosilicate bottles. The POCIS used a hydrophobic/lipophilic balanced polymer encased in two porous polyethersulfone membranes (47 mm) to accumulate polar organic compounds on media in contact with the stream water (Ahrens et al., 2015; Alvarez et al., 2005; Charlestra et al., 2012) (Figure S1). The POCIS were produced by Environmental Sampling Technologies (Patent #6,478,961 B2) and were deployed inside a polyvinyl chloride cage to protect the membranes from puncture and were suspended in the stream with braided steel cable. During these relatively short deployment periods, we assume the POCIS accumulate neonicotinoids at a rate proportional to stream water concentrations, the time period of deployment (*t_d_*) and a compound‐specific empirical constant called the POCIS sampling rate (*R_S_*) (Alvarez, 2010). The mass of neonicotinoid accumulated by the POCIS (M_NNI_) can be expressed as follows:

(1) $$M_{\mathrm{NNI}}=Ct_{d}R_{S},$$

where after retrieval of the POCIS, the mass of accumulated neonicotinoid can be converted into a time‐averaged stream concentration (*C*). Aqueous discrete samples and POCIS were stored on ice and transferred to the Water and Environmental Analysis Laboratory at the University of Wisconsin–Stevens Point, where they were refrigerated at 4°C. The aqueous samples were processed using solid‐phase extraction techniques and extracts analyzed via high‐performance liquid chromatography and tandem mass spectrometry (Agilent 6430 LC/MS/MS, Tables S1–S5). POCIS were disassembled, the sorption media removed, and neonicotinoids extracted using methanol and analyzed by LC/MS/MS. Table 2 lists the limits of detection for each neonicotinoid with the range of sampling rates based on previous studies (Ahrens et al., 2015; Poulier et al., 2015; Sultana et al., 2018; Taylor et al., 2020). POCIS field replicates were conducted at a 20% frequency rate (four replicates) and showed an average relative percent difference from 16% to 32% (Table S6). Two POCIS were designated as blanks where they were treated similarly to field‐deployed POCIS. Five blank discrete samples were processed along with surface water samples.

**TABLE 2:** Stream concentrations of neonicotinoids.

Site number	Thiamethoxam (ng L^−1^)			Clothianidin^a^ (ng L^−1^)			Imidacloprid^a^ (ng L^−1^)		
	Discrete		POCIS^b^ time average	Discrete		POCIS^b^ time average	Discrete		POCIS^b^ time average
	Aug.	Sept.		Aug.	Sept.		Aug.	Sept.	
1	<1.5	<1.5	<0.05	<1.5	<1.5	<0.05	<2.3	<2.3	<0.08
2	<1.5	<1.5	<0.05	<1.5	<1.5	<0.05	<2.3	<2.3	0.13 (0.1–0.2)
3	2.1	2.6	1.33 (0.8–1.6)	5.7	5.2	1.87 (1.6–2.7)	2.6	1.9	1.75 (1.7–2.8)
4	1.7	3.8	0.53 (0.3–0.6)	11.1	11.3	2.03 (1.8–3.0)	8.2	7.5	1.37 (1.3–2.2)
5	6.8	7.5	1.96 (1.2–2.4)	7.4	4.9	1.55 (1.4–2.3)	<2.3	<2.3	0.11 (0.1–0.2)
6	<1.5	<1.5	<1.5	<1.5	<1.5	0.52	<2.3	<2.3	<0.08
7	<1.5	<1.5	0.15 (0.09–0.18)	2.3	1.3	0.64 (0.6–0.9)	<2.3	<2.3	0.08 (0.08–0.13)
8	2.9	2.8	3.36 (2.0–4.0)	1.8	1.7	1.55 (1.4–2.3)	<2.3	<2.3	0.56 (0.54–0.90)
9	9.2	3.6	11.0 (6.6–13.2)	14.3	5.3	9.21 (8.1–13.5)	<2.3	<2.3	1.98 (1.90–3.17)
10	<1.5	<1.5	0.76 (0.46–0.91)	4.0	3.9	2.65 (2.3–3.9)	<2.3	<2.3	0.27 (0.26–0.43)
11	<1.5	<1.5	<1.5	<1.5	<1.5	0.39	<2.3	<2.3	<0.08
12	5.2	8.0	4.3 (2.6–5.2)	18.8	16.1	9.88 (8.7–14.5)	**40.9**	**31.6**	**21.4** (**20.5–34.2**)
13	197	184	130 (78–156)	**74.4**	**77.7**	39.5 (34.8–**57.9**)	**21.5**	**22.5**	**11.6** (**11.1**–**18.6**)
14	83.0	115	69.0 (41.4–82.8)	**61.1**	47.8	33.9 (29.8–49.7)	**33.2**	**24.0**	**21.9** (**21.0**–**35.0**)
15	101	127	116 (70–139)	37.6	34.7	32.0 (28.1–46.9)	**21.2**	**22.2**	**19.8** (**19.0**–**31.7**)
16	286	234	320 (192–384)	42.8	24.6	12.3 (10.8–18.0)	**24.7**	**19.0**	6.91 (6.63–**11.1**)
17	95.3	79.9	63.9 (38.3–76.7)	25.8	15.6	14.3 (12.6–21.0)	**16.8**	**11.1**	9.39 (9.0–**15.0**)
18	74.1	66.2	52.9 (31.7‐63.5)	**59.2**	42.9	33.5 (29.5–49.1)	**30.2**	**24.8**	**19.4** (**18.6**–**31.0**)
19	251	212	207 (124–248)	**62.5**	37.0	37.2 (32.7–54.6)	**48.5**	**36.0**	**29.9** (**28.7**–**47.8**)
20	411	397	328 (197–394)	**147**	**104**	**74.3** (**65.4**–**109.0**)	**27.4**	**23.8**	**16.4** (**15.7**–**26.2**)
Limit of detection	1.5		0.05	1.5		0.05	2.3		0.08
Detection Frequency	70%		80%	80%		90%	55%		85%
Conc range (ng L^−1^)		0.15–411			0.52–147			0.08–48.5	

*Note*: Bold values denote exceedance of a concentration of concern.

Abbreviations: EPA, Environmental Protection Agency; POCIS, polar organic chemical integrative sampler.

^a^ Concentrations in bold exceed the US EPA Chronic Aquatic Life Benchmark (Table 1).

^b^ Using a POCIS *R_S_* (and range) in L day^−1^ for thiamethoxam: 0.12 (0.10–0.20); clothianidin: 0.22 (0.15−0.25); and imidacloprid: 0.24 (0.15–0.25) based on literature values (Ahrens et al., 2015; Poulier et al., 2015; Sultana et al., 2018; Taylor et al., 2020).

### 2.3 Hydrology and land cover

Sampling locations (Figure 1) were on lower order streams and selected to provide a range of contributing land areas and agricultural land cover. Ten years of streamflow monitoring at these locations show average flow ranging from 0.05 to 1.5 m^3^ s^−1^ (Flow Tracker 2, Sontek). In addition, continuous flow measurement during the deployment period was available from six USGS streamgages in the study area (Figure 1). The land area contributing groundwater discharge to each site was determined using a groundwater flow model (MODFLOW) coupled with a particle‐tracking model (MODPATH) (Kraft & Mechenich, 2010; Kraft et al., 2012). The flow model used 200 m × 200 m grid cells and two layers to simulate the relatively conductive outwash and tills with an average hydraulic conductivity of 25 m day^−1^ over a less conductive bedrock. The model had been calibrated by adjusting hydraulic conductivity to match measured average water table and streamflow conditions. We approximated the higher water table and streamflow during the study period by increasing the recharge to match the streamflow measured at the sample sites (Figure S2). Forward particle tracking and a porosity of 0.3 were used to identify the land area where groundwater originates and the groundwater travel time to the stream for each sampling location. Four particles were assigned to each model grid cell, resulting in travel time for each 100 m × 100 m. The land cover in the area contributing to each sample site was characterized using the geospatial landcover grids from Wiscland 2.0 (WDNR, 2016). Agricultural landcover in the Wiscland 2.0 database is divided into agricultural rotations based on 5 years (2008–2012) of the United States Department of Agricultural Statistics Service Cropland Data Layers.

Stream concentrations were related to land cover by assuming the stream concentration was the volumetric average of groundwater originating from across the groundwater contributing area (B. A. Browne & Guldan, 2005; Modica et al., 1997). Because these neonicotinoids have been used for less than 25 years, only those portions of the groundwater contributing area with a travel time less than that usage time would have neonicotinoids in the groundwater they deliver to the stream. We divided the groundwater contributing area into travel time zones using the groundwater flow modeling (Figure S3). We assumed travel time in the unsaturated zone was relatively short and travel time in the aquifer was not influenced by sorption (Kraft & Stites, 2003; Kruisdijk et al., 2022; Kung, 1990; Pietrzak et al., 2022; Yost et al., 2019) (Table S7). By assuming a uniform annual groundwater recharge rate across the entire groundwater contributing area and a step increase in neonicotinoid concentration at the start of the usage period, the contribution to the current stream concentration from a land cover *i* in a travel time zone is calculated from the area of that land cover relative to the entire groundwater contributing area, *A_i,t_*/*A_T_*, with the possibility of first‐order degradation included with a rate constant (*k*) and travel time (t) for that zone. We used travel time zones of 1–5 years and the midpoint time for each zone. The current stream concentration (C) is modeled by summing those contributions for all neonicotinoid‐contributing landcovers over the period of neonicotinoid usage:

(2) $$C=\sum_{i}^{\mathrm{land}\;\mathrm{covers}}C_{0i}\left(\;\sum_{t}^{\mathrm{usage}\;\mathrm{time}}\left(\frac{A_{i,t}}{A_{T}}\;e^{-kt}\right)\right),$$

where *C*_0_ *_i_* is a flow‐weighted concentration entering groundwater beneath a land cover. This model simplifies the description of neonicotinoid movement to groundwater beneath a field as an average concentration when the period of usage begins. While neonicotinoid concentrations in water percolating through the soil profile vary throughout the year and between years with variations in neonicotinoid use rates, application timing and water movement change (Huseth & Groves, 2014), this model was developed to compare neonicotinoid transfer to streams at a watershed scale where a land cover system combines flow from multiple fields over multiple years. If neonicotinoid degradation in the aquifer can be ignored, Equation (2) can be simplified to weighting the groundwater concentrations beneath the land covers by their land area within a travel time equal to the usage period:

(3) $$C_{\left(k=0\right)}=\sum_{i}^{\mathrm{land}\;\mathrm{covers}}C_{0i}\frac{A_{i}}{A_{T}},$$

where *A_i_*/*A_T_* is the fraction of the full groundwater contributing area that is occupied by a land cover within the travel time corresponding to the neonicotinoid‐use period prior to stream sampling. We approximated the use period in the WCS as 25 years for imidacloprid, 15 years for thiamethoxam, and 10 years for clothianidin (Figure S4).

The relationship between land cover systems and the neonicotinoid stream concentrations was examined with the correlation between the stream concentrations and the ratio of that land cover to the total groundwater contributing area assuming the neonicotinoids are conservative or are reduced by a first‐order degradation rate in the aquifer. Spearman rank correlations were calculated using the R psych package corr.test function (R Core Team, 2013; Revelle, 2026).

Stream concentrations and the land cover distributions were used to calculate the corresponding average groundwater concentrations beneath a land cover, *C*_0,_ *_i_* (Equation 2 or 3). These concentrations were compared to groundwater concentrations measured in field‐edge monitoring wells within the WCS (Bradford et al., 2018; Huseth & Groves, 2014; Senger et al., 2019). Monitoring well results from 2008 to 2025 were used (Ken Potrykus,Wisconsin Department of Agriculture, Trade and Consumer Protection, personal communication, 2026), during which imidacloprid, thiamethoxam, and clothianidin were all used in the WCS. These samples were collected from monitoring well nests with multiple wells having short (1.5 m or 3 m) screened intervals. Only the upper 3 m of screened intervals from each location were used to provide samples most representative of field‐edge groundwater. Results were pooled from screened intervals in the same well nest. Groundwater concentrations associated with land covers that were estimated from the stream concentrations were used to calculate the corresponding unit‐area export to groundwater (Kraft et al., 2008) where the flow‐weighted concentration, *C*_0,_ *_i_* (µg m^−3^ or ng L^−1^), in groundwater beneath a land cover system *i* is equal to the average annual unit‐area mass loss to groundwater, *X_i_* (µg m^−2^ year^−1^) divided by the annual water moving into groundwater, *D_R_* (m year^−1^).

(4) $$C_{0,i}=\frac{X_{i}}{D_{R}}.$$

## 3 RESULTS AND DISCUSSION

### 3.1 Neonicotinoid concentrations in streams

Results from the discrete and POCIS samples are summarized in Table 2 by their frequency of detection and concentration range. POCIS sampling increased the frequency of detection for the neonicotinoids because the estimated detection limit with a POCIS is 30 times lower than with a discrete aqueous sample. Neonicotinoid concentrations exceeded chronic EPA ALBs for imidacloprid at nine of 20 sites and for clothianidin at five of 20 sites.

The concentrations were similar in the two discrete samples and the time‐averaged concentrations calculated for the POCIS at each sampling location (Table 2) consistent with the groundwater‐dominated stream hydrology in the WCS. During the POCIS deployment period, there was rainfall in parts of the study area and hydrographs from USGS streamgages show streamflow response during these periods for several days near the middle of the deployment period (Figure 1). The relatively small differences between the two discrete samples and the POCIS results are similar to other studies of streamflow chemistry in the WCS (Hindall, 1978) and consistent with increases in flow arising from additional groundwater inflow (Miller et al., 2017). The results also show that both discrete sampling and POCIS can effectively monitor neonicotinoids in surface water or groundwater with detection limits far below regulatory standards or US EPA ALBs. Discrete sampling followed by solid‐phase extraction of water samples combined with advanced instrumentation such as liquid chromatography–mass spectrometry can provide detection limits of <5 ng L^−1^. Time‐weighted average concentrations of neonicotinoids determined with POCIS can provide detection limits that are <0.1 ng L^−1^.

### 3.2 Land cover in the groundwater contributing areas

**FIGURE 2:**
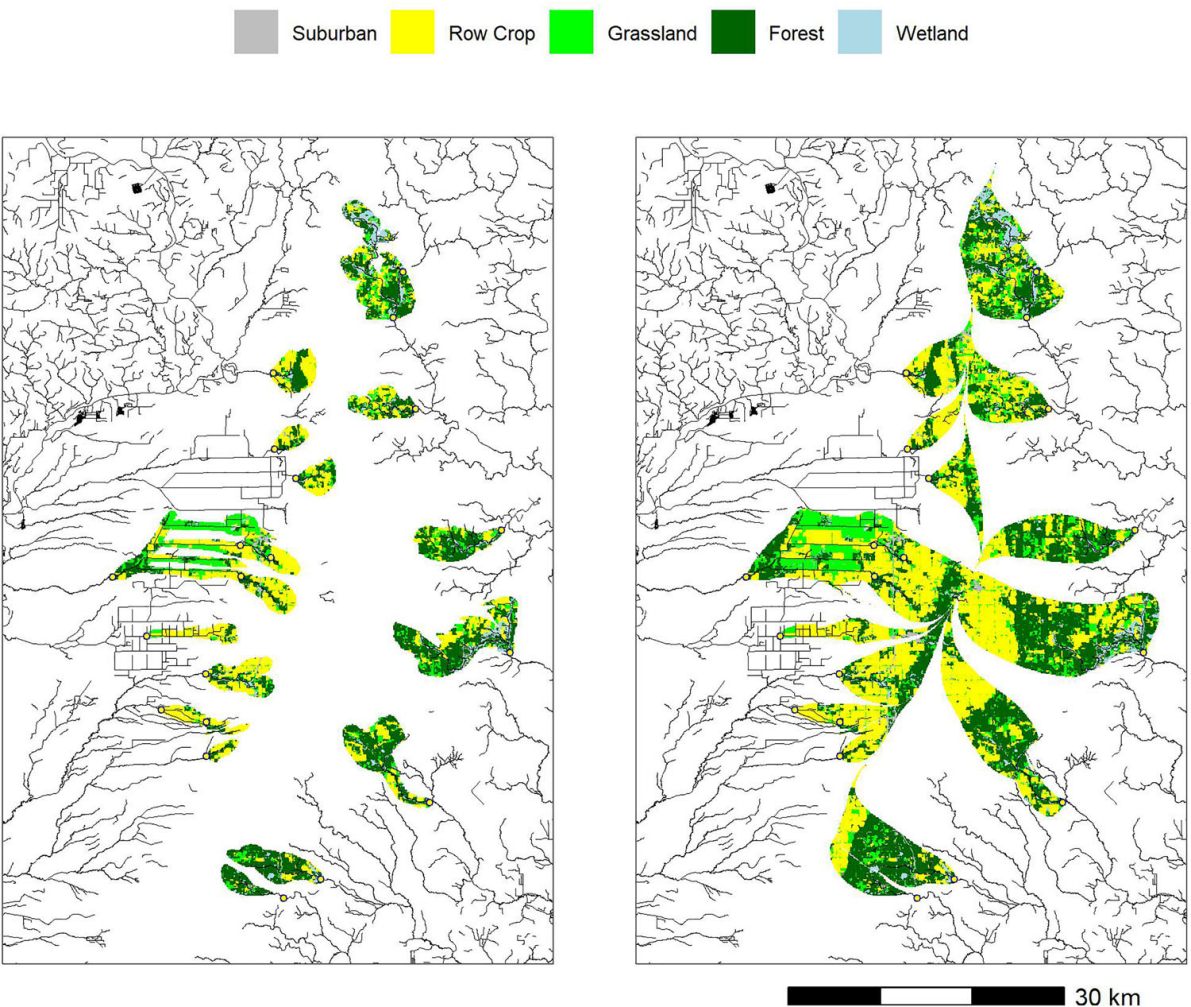
Groundwater contributing areas and land cover for the sample sites within a 20‐year groundwater travel time (left) and the full groundwater area (right). Row crop includes potato/vegetable, dairy, cranberry, and corn/grain.

**FIGURE 3:**
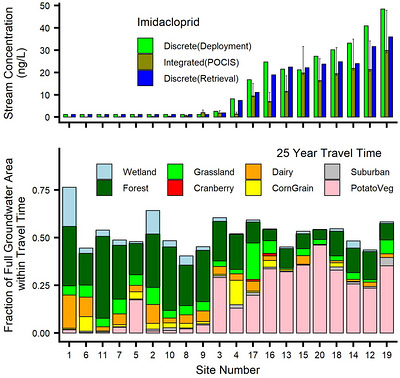
Concentrations of imidacloprid at sites ranked by concentration (top) compared to the area of land cover in the 25‐year travel time relative to the full groundwater area (lower). Polar organic chemical integrative sampler (POCIS) sampling rate (*R_S_*) ranges in Table 2 were used to create concentration error bars.

**FIGURE 4:**
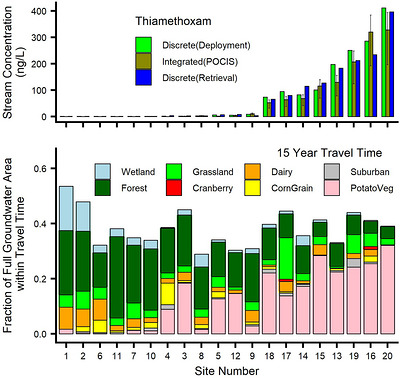
Concentrations of thiamethoxam at sites ranked by concentration (top) compared to the area of land cover in the 15‐year travel time relative to the full groundwater area (lower). Polar organic chemical integrative sampler (POCIS) sampling rate (*R_S_*) ranges in Table 2 were used to create concentration error bars.

**FIGURE 5:**
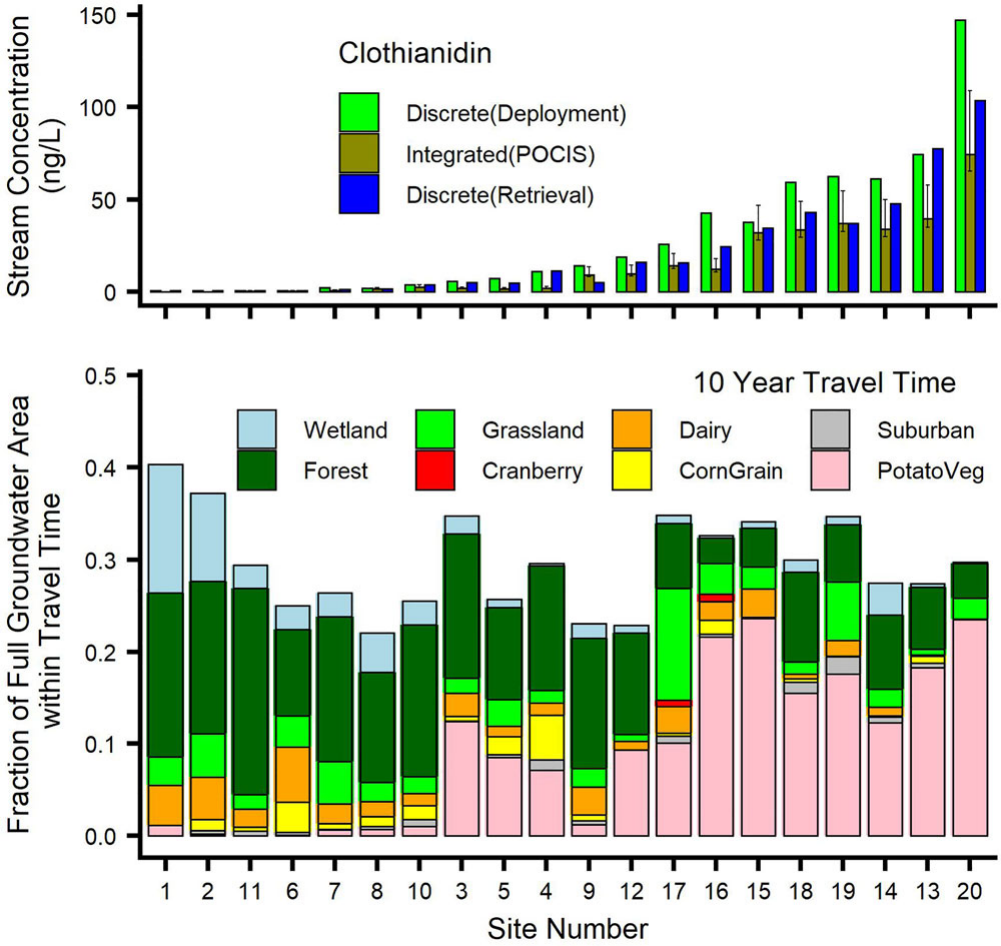
Concentrations of clothianidin at sites ranked by concentration (top) compared to the area of land cover in the 10‐year travel time relative to the full groundwater area (lower). Polar organic chemical integrative sampler (POCIS) sampling rate (*R_S_*) ranges in Table 2 were used to create concentration error bars.

The land area that contributes groundwater to the stream upstream of each sample site is shown in Figure 2 for the 20‐year and full groundwater contributing areas for each sample location. The full groundwater contributing areas ranged from 4 to 160 km^2^. Streamflow originates from the entire groundwater contributing area but only those areas with a groundwater travel time to the stream within approximately 10–25 years prior to the sampling are expected to be a source of neonicotinoids in that streamflow. The distribution of travel times within the contributing areas was used to develop a travel time distribution for each sample site (Figure S3). In most cases, only one‐third to two‐thirds of the water entering the streams was young enough at the time of sampling to be contributing neonicotinoids to the stream. Higher imidacloprid concentrations in the stream correlate with a higher density of potato/vegetable agriculture within a 25‐year travel time to the stream relative to the full groundwater contributing area (Figure 3; Table 3). Similar results were found within a 15‐year travel time for thiamethoxam (Figure 4; Table 3) and within a 10‐year travel time for clothianidin (Figure 5; Table 3). High correlations between the potato/vegetable land cover in the groundwater contributing area and neonicotinoid concentrations in the stream were found with and without assuming degradation in the aquifer reflecting the similarity of land cover density in the different travel time zones. The strong positive correlation between neonicotinoid concentrations and potato/vegetable agriculture is consistent with the findings of previous research in the study area (Huseth & Groves, 2014). This cropping system uses rotations of potato with beans (*Phaseolus vulgaris* L.), corn (*Zea mays* L.), and other vegetable crops (Bradford et al., 2018; Heineman & Kucharik, 2022; Huseth & Groves, 2014). Potatoes and vegetables have relatively shallow root zone depths and in this region are usually irrigated (Sanford & Panuska, 2019). Neonicotinoid application rates in these crops could range from 50 to 500 g ha^−1^ year^−1^ (Huseth & Groves, 2014; USDA‐NASS, 2023; Woodward et al., 2022).

**TABLE 3:** Spearman rank correlations for neonicotinoid POCIS concentrations with land cover density^a^ in the groundwater contributing area.

	Imidacloprid 25 year travel time		Thiamethoxam 15 year travel time		Clothianidin 10 year travel time	
Land cover	Assuming no degradation	Assuming 3 year half‐life	Assuming no degradation	Assuming 3 year half‐life	Assuming no degradation	Assuming 3 year half‐life
Wetland	−0.50*	−0.53*	−0.66**	−0.70***	−0.67**	−0.70***
Forest	−0.75***	−0.63**	−0.90***	−0.83***	−0.80***	−0.73***
Grassland	−0.37	−0.10	−0.15	0.04	−0.22	−0.09
Cranberry	0.11	0.11	0.35	0.35	0.17	0.17
Dairy	−0.61**	−0.40	−0.52*	−0.39	−0.62**	−0.53*
Corn/grain	−0.54*	−0.47*	−0.35	−0.28	−0.39	−0.29
Potato/vegetable	0.83***	0.81***	0.89***	0.90***	0.85***	0.86***
Suburban	0.34	0.30	0.12	0.26	0.33	0.42

^a^ Ratio of the land cover in the usage period travel time portion of the groundwater contributing area relative to the full groundwater contributing area adjusted for any first‐order loss (Equation 2).

***p < 0.001, **p < 0.01, *p < 0.05.

The neonicotinoid stream concentrations show only a weak or negative correlation between the dairy and corn/grain agricultural land cover systems in the groundwater contributing area. Although these agricultural systems are likely to be using neonicotinoids, particularly as seed treatment for corn and soybeans (*Glycine max* (L.) Merr.), dairy also includes forage crops such as alfalfa (*Medicago sativa* L.) for multiple years within a field rotation. Furthering complicating the relationship is that the quantity of neonicotinoids for seed treatments may have been increasing in the decade prior to our monitoring (Tooker et al., 2017). Overall, the agricultural landcover within our study areas was dominated by potato/vegetable agriculture, making it difficult to use these results to evaluate other agricultural systems.

### 3.3 Export to groundwater and future stream concentrations

**FIGURE 6:**
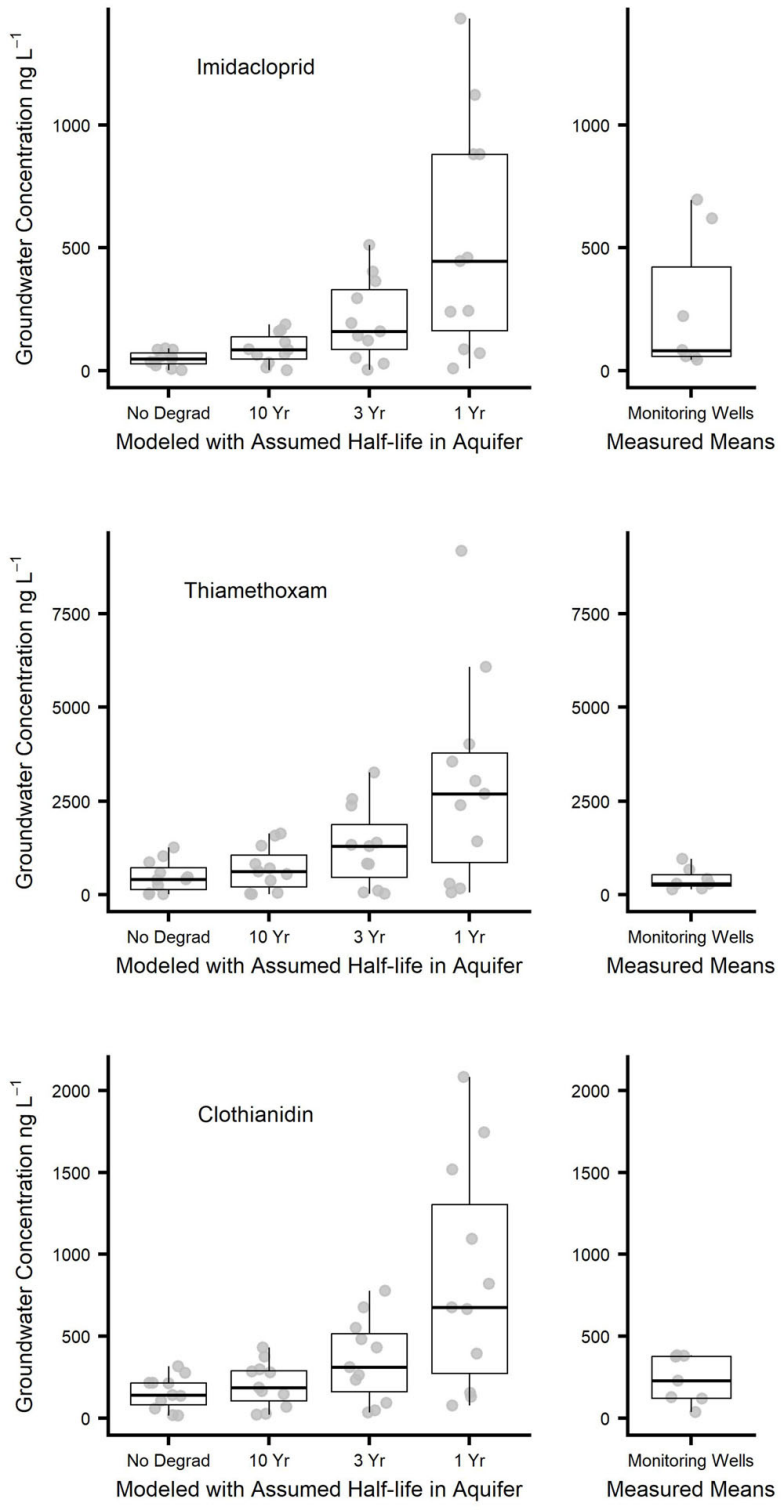
Modeled concentrations of imidacloprid (upper), thiamethoxam (middle), and clothianidin (lower) in groundwater near potato/vegetable fields from stream concentrations, assuming different rates of degradation in the aquifer (left) compared to mean concentrations measured in field‐edge groundwater monitoring wells (right). Using sites with a large fraction of potato/vegetable land cover (sites 3, 5, and 12–20).

The measured stream concentrations and adjusted land cover fractions were used to calculate a corresponding groundwater concentration (*C*_0_) (Equation 2) from the potato/vegetable land cover (Figure 6). This groundwater concentration represents an average field‐edge concentration associated with that land cover. This assumed the potato/vegetable system was the only contributor of neonicotinoids to the stream concentrations and used only those sites where the potato/vegetable land cover was more than 65% of the total agriculture and suburban land cover within the usage period travel time (sites 3, 5, and 12–20). If there was no degradation in the aquifer, these groundwater concentrations would correspond to stream concentrations adjusted by the potato/vegetable land cover fraction of that groundwater contributing area (Equation 3). If there was degradation in the aquifer, the corresponding groundwater concentrations would be higher as they account for loss during transport within the aquifer (Equation 2). Figure 6 shows how assuming a 1‐year half‐life in the aquifer leads to field‐edge groundwater concentrations that are approximately fivefold to 10‐fold higher than those without degradation in the aquifer. The similarity between the modeled groundwater concentrations that assume a long half‐life or no degradation in the aquifer and the mean values measured in field‐edge monitoring wells supports assuming only a relatively slow rate of neonicotinoid degradation in the aquifer (Figure 6). Slow degradation or persistence in the aquifer is similar to previous research on atrazine and chloroacetanilide herbicides in groundwater (Farlin et al., 2017; Kraft et al., 2008). Neonicotinoids have been shown to degrade slowly in surface water and soil with half‐lives ranging from several weeks to several years (Kruisdijk et al., 2022; Leiva et al., 2015; Pietrzak et al., 2020), and it is reasonable to assume neonicotinoids will be more persistent in groundwater where they are not exposed to sunlight, temperatures are low, and microbial activity is likely lower. Additional research into the long‐term persistence of neonicotinoids in groundwater is necessary as the field‐edge groundwater exhibits large variations in neonicotinoid concentrations (Bradford et al., 2018; Huseth & Groves, 2014; Senger et al., 2019). Our comparison (Figure 6) uses the mean value for each well collected over 17 years of monitoring (2008–2025; Figures S5–S10; Tables S8–S10), but concentrations in these wells range from non‐detected to more than 5000 ng L^−1^ and a similarly wide range has been measured in soil percolate within potato fields (Huseth & Groves, 2014).

We used our modeled groundwater concentrations from the potato/vegetable agricultural land cover to calculate a corresponding mass loss rate to groundwater (Equation 4). Assuming the annual recharge rate of 0.33 m year^−1^ from our groundwater modeling and no degradation in the aquifer, these stream concentrations would be explained by a mean annual loss rate from potato and vegetable landcover of 16 (range 0.2–30) µg m^−2^ year^−1^ (0.16; range 0.002–0.3 g ha^−1^ year^−1^) for imidacloprid, 158 (range 2.4–415.0) µg m^−2^ year^−1^ (1.6; range 0.02–4.1 g ha^−1^ year^−1^) for thiamethoxam, and 51 (range 5.0–104.4) µg m^−2^ year^−1^ (0.5; range 0.05–1.0 g ha^−1^ year^−1^) for clothianidin. This could be a small percentage of the possible annual application rate to fields (e.g., 50–500 g ha^−1^ year^−1^), but still lead to stream concentrations that can exceed the ALB for both imidacloprid and clothianidin and total neonicotinoid concentrations that can exceed 35 ng L^−1^.

Delivery of only a small fraction of applied neonicotinoid to streams through groundwater is consistent with several in‐field mass balance studies. For example, in a 9‐month study of neonicotinoids in tile drainage from treated seeds, 0.5% and 1.2% of imidacloprid and thiamethoxam, respectively, were recovered in the drainage (Wettstein et al., 2016). Similarly, an 8‐month mass balance of clothianidin‐treated corn recovered 0.33% in tile drainage (Alford & Krupke, 2019). Although our research is at a much larger spatial and temporal scale, and we can only assume likely application rates, comparing our mean delivery rate estimates of 0.16–1.6 g ha^−1^ year^−1^ with annual application rates that could range from 50 to 500 g ha^−1^ suggests a similarly low fraction of applied neonicotinoids are delivered from groundwater in stream baseflow.

**FIGURE 7:**
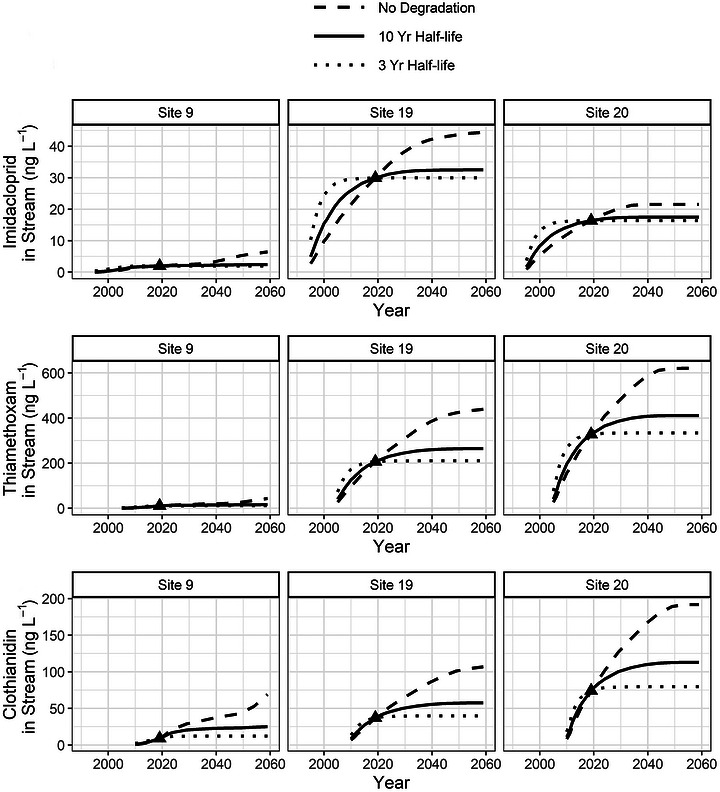
Examples of back‐ and forecasted neonicotinoid concentrations over time for several stream sites, assuming uniform export rates to groundwater based on current stream measurements and different aquifer degradation half‐life scenarios. The triangle marker shows the measurements for this study in year 2019.

The stream concentrations we measured exceed the ALB for imidacloprid and clothianidin in several locations and an important question for pesticide management is how stream concentrations may change in the future. Assuming that the average export rate to groundwater from the potato and vegetable land cover does not change within each groundwater contributing area for the sampling locations, future concentrations can be projected. Figure 7 shows future concentrations will be influenced by the rate of degradation in the aquifer. If half‐lives in the aquifer are 3 years or less, the neonicotinoid concentrations in the stream are likely already controlled by degradation in the aquifer and in the absence of changes in the transfer to groundwater, the stream concentrations will remain relatively constant. By contrast, if the neonicotinoids are more persistent in the aquifer with half‐lives of 10 years or greater, the neonicotinoid concentrations will continue to increase and could more than double at some locations in the next several decades.

There are several sources of uncertainty using stream concentrations and groundwater modeling to provide estimates of neonicotinoid loss at the field‐scale. Our monitoring occurred during a time when baseflow groundwater discharge to the streams was higher than average. The water delivery rate at the time of sampling was approximately 25%–30% higher than the long‐term average and reflects several consecutive years of above average precipitation. This could lead to variations in the acquisition and movement of neonicotinoids within the soil profile and to groundwater but because the stream discharge represents several decades of neonicotinoids in groundwater recharge, our results should average year‐to‐year variations in movement from soil profile to groundwater. Changes in baseflow discharge to the stream reflect changes in water table elevation in the aquifer. Although this results from year‐to‐year changes in groundwater recharge rate across the contributing area, it also influences the rate at which groundwater that has been in the aquifer for decades is discharged to the stream. If the higher baseflow does not alter the distribution of travel time ages in the streamflow, the stream concentrations would not change but the unit‐area neonicotinoid stream delivery rates we calculate could be 25%–30% higher than the long‐term average. Other sources of uncertainty include assuming there was no sorption in the aquifer. Sorption will slow neonicotinoid transport. Because of the low organic carbon content of the aquifer and the high water solubility of the neonicotinoids, only weak sorption is expected, but if there is more sorption, perhaps resulting from higher organic carbon in some areas, the neonicotinoids would travel slower than the water in the aquifer and the current stream concentrations will reflect contributions from a smaller part of the contributing area than was assumed. We also assumed any changes to the neonicotinoid concentration during transport in the stream were minor as this time is likely relatively short (e.g., hours to days). Any reduction in neonicotinoid concentrations in the stream would lead to our underestimating the loss from land to stream. Finally, a major challenge to understanding the delivery of neonicotinoids at the field‐scale is the changing nature of their use. For example, the time period of application relevant to this study included the large increase in neonicotinoids use as a seed treatment. Future monitoring will be necessary to see how those changes will move through the groundwater system.

## 4 CONCLUSIONS

Neonicotinoids were pervasive in surface water throughout the WCS where the land that contributes water to the stream included potato and vegetable agriculture. This research shows how neonicotinoid concentrations in stream baseflow can result from land application of these insecticides and delivery in groundwater of a relatively small percentage of those likely applied. Stream concentrations of thiamethoxam were higher than the other neonicotinoids, but concentrations of thiamethoxam did not exceed the chronic EPA ALB at any of the study sites. Imidacloprid exceeded the chronic EPA ALB at nine of 20 sites. Clothianidin concentrations exceeded the ALB at five of 20 sites. Our total neonicotinoid concentrations exceeded the Morrissey et al. chronic benchmark concentration of 35 ng L^−1^ at nine out of 20 (45%) sites monitored. Comparison of modeled groundwater concentrations from potato/vegetable land cover with measured field‐edge concentrations supports a relatively slow degradation of neonicotinoids in the aquifer, and the stream concentrations are consistent with neonicotinoid delivery from the potato/vegetable agricultural system up to 4 g ha^−1^ year^−1^. As the water originating further from the stream adds to the neonicotinoid load in baseflow, we project that the concentrations in the stream will increase if these compounds behave conservatively in the aquifer.

## AUTHOR CONTRIBUTIONS

**William M. DeVita**: Conceptualization; formal analysis; funding acquisition; investigation; methodology; project administration; writing—original draft. **Paul M. McGinley**: Conceptualization; funding acquisition; investigation; methodology; software; writing—original draft.

## CONFLICT OF INTEREST STATEMENT

The authors declare no conflicts of interest.

## Supporting information



Supplemental material
