# Supplementary material for: Groundwater delivery of neonicotinoids to streams
Source: J Environ Qual. 2026 Aug 16;55(4):e70234. doi: 10.1002/jeq2.70234 (PMC13478542; doi:10.1002/jeq2.70234)
Supplement: Supplementary file 1 [file JEQ2-55-0-s001.pdf]

Supplemental Information for:

Groundwater Delivery of Neonicotinoids to Streams

*William M. DeVita and Paul M. McGinley**

Center for Watershed Science and Education, College of Natural Resources, University of Wisconsin-Stevens Point, Stevens Point WI 54481, USA

*Corresponding Author

E-mail: paul.mcginley@uwsp.edu

Table of Contents

S1. POCIS (Polar Organic Compound Integrative Samplers) 3
S2. LC/MS/MS Acquisition Method for Neonicotinoid Insecticides 5
S3. Calibration Summary 7
S4. Groundwater Flow Modeling 9
S5. Chemical Characteristics of the Neonicotinoids 13
S6. Neonicotinoid Use History in the Wisconsin Sand Plain 14
S7. Field-Edge Groundwater Monitoring Wells 15
References 22

Tables

Table S1. Agilent 6430 Electrospray Ionization Source Parameters 5
Table S2. Scan Parameters. All Ions Monitored in Positive Ion Mode 5
Table S3. Agilent 1200 High Performance Liquid Chromatograph Parameters 5
Table S4. Method Detection Limit Study for Neonicotinoids in Aqueous Samples 6
Table S5. Recovery of Surrogate Standards 7
Table S6. Relative Percent Differences of Field Deployed POCIS 8
Table S7. Chemical Characteristics of the Neonicotinoids 13
Table S8. Imidacloprid Field-Edge Monitoring Well Summary 17
Table S9. Thiamethoxam Field-Edge Monitoring Well Summary....19
Table S10. Clothianidin Field-Edge Monitoring Well Summary....21

Figures

Figure S1. POCIS Diagram 2
Figure S2. Measured and Modeled Streamflow 11
Figure S3. Groundwater Travel Time Distributions at the Sampling Locations 12
Figure S4. History of neonicotinoid use in central Wisconsin 14
Figure S5. Imidacloprid concentrations in field-edge monitoring wells 15
Figure S6. Imidacloprid concentrations in field-edge monitoring wells over time 16
Figure S7. Thiamethoxam concentrations in field-edge monitoring wells...........................18
Figure S8. Thiamethoxam concentrations in field-edge monitoring wells over time...........18
Figure S9. Clothianidin concentrations in field-edge monitoring wells...............................20
Figure S10. Clothianidin concentrations in field-edge monitoring wells over time............20

S1. POCIS Passive Samplers

Polar Organic Compound Integrated Samplers (POCIS) are passive samplers used to measure a time-averaged concentration during a deployment period. Figure S1 shows the design of the POCIS used in this study. The POCIS used a hydrophobic/lipophilic balanced (HLB) polymer encased in two porous polyethersulfone membranes (47 mm). The POCIS were produced by Environmental Sampling Technologies, St. Joseph, MO, USA (Patent #6,478,961 B2). They were deployed inside a PVC cage to protect the membranes from puncture and were suspended in the stream with braided steel cable. Membranes and polymeric media were held together with stainless-steel rings.

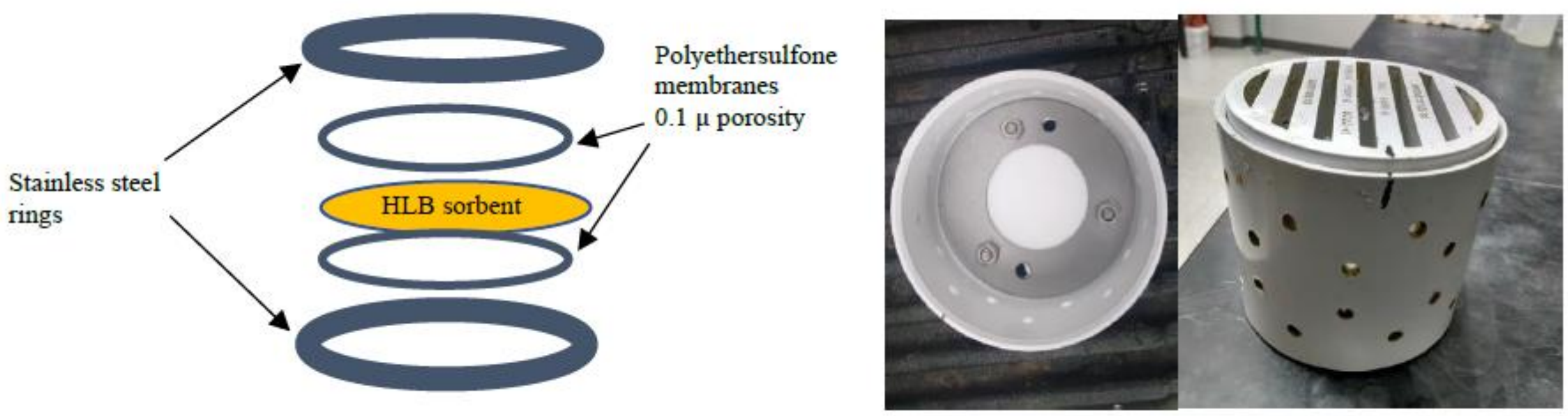

Figure S1. POCIS diagram (left). Inserted into protective PVC cage (center and right image).

POCIS passive samplers performed satisfactorily considering the many steps in the entire process of deployment, retrieval, removal of the sorbent, extraction, and analysis. Field replicates were conducted at a 20% frequency rate (four replicates) throughout the study. Relative percent difference (RPD) of the three detected NNI averaged from 16-32%. While RPD is an often-used metric for precision, low concentrations will often record greater differences. However, it is noted that even at higher concentrations, RPD varied. This could be due to an accumulation of fine sediment on the surface of the membrane that might limit water contact with the media,

media that escaped the membrane during deployment or analytical process, reduced extraction efficiency, or interferences in the LC/MS process.

Unlike other passive samplers such as semi-permeable membrane devices, the POCIS do not typically rely upon a performance reference compound (PRC). The PRC can provide an estimate of loss of an analyte after it has been accumulated by the passive sampler. With media-based passive samplers such as the POCIS, loss of an analyte is governed by the surrounding matrix (water in this case) and its ability to remove the analyte from the media. Typically, water is not a strong enough solvent to remove compounds from the HLB media in a POCIS. For this reason, PRCs were not used, and matrix spike recoveries were based upon adding a NNI mixture to clean, unexposed HLB media, then extracted. Interferences were minimal in these spiked POCIS and recoveries were typically >80%.

S2. LC/MS/MS Acquisition Method for Neonicotinoid Insecticides

Table S1. Agilent 6430 Electrospray Ionization Source Parameters

Gas Temperature - ºC	350
Gas Flow - liters/minute	12
Nebulizer Pressure (psi)	60
Capillary Voltage	4000

Table S2. Scan parameters. All Ions Monitored in Positive Ion Mode

Analyte		Precursor	Product	Dwell	Frag.	Collision	Cell Accel.
		Ion	Ion		energy	energy	energy
Thiamethoxam-d3	Int.	295.0	214.0	30	72	5	4
	Std.	295.0	184.0	30	72	20	2
Thiamethoxam	target	292.0	211.0	30	72	5	4
		292.0	181.0	30	72	20	2
Imidacloprid- d4	Surr.	260.1	213.1	30	76	18	1
	Std.	260.1	179.1	30	76	18	1
Imidacloprid	target	256.1	209.1	30	76	18	1
		256.1	175.1	30	76	18	1
Clothianadin	target	250.0	169.1	30	66	10	1
		250.0	131.1	30	66	10	1
Acetamiprid	target	223.0	126.0	30	95	16	1
		223.0	56.1	30	95	16	1
Dinotefuran	target	203.1	129.1	30	63	8	2
		203.1	113.1	30	63	8	2

Table S3. Agilent 1200 high performance liquid chromatograph parameters

Column	Agilent Eclipse Plus C18, 1.8µm, 2.1 x 100 mm			
Solvent A Composition	15 mM acetic acid			
Solvent B Composition	15 mM acetic acid in methanol			
Column Temperature	60 (ºC)			
Injection Volume	0.020 mL			
HPLC Gradient Timetable	Time (min)	%A	%B	Flow (ml/min)
	initial	90	10	0.300
	6	90	10	0.300
	10	20	80	0.300
	12	20	80	0.300
	14	90	10	0.300

Table S4. Method Detection Limit Study for Neonicotinoids in Aqueous Samples using Solid Phase Extraction (250 mL sample, fortified at 5 ng/L each neonicotinoid insecticide)

(Note: this study was conducted prior to the US EPA changing the process for determining a laboratory's method detection limits)

	Thiamethoxam		Clothianidin		Imidacloprid	
	[Sample] ng/L	% Recovery	[Sample] ng/L	% Recovery	[Sample] ng/L	% Recovery
	4.75	95.1	4.98	99.5	5.99	119.9
	5.24	104.8	5.33	106.7	6.42	128.5
	4.75	94.9	5.22	104.3	6.27	125.3
	6.13	122.6	6.32	126.4	6.56	131.3
	5.83	116.5	4.83	96.5	4.98	99.5
	5.67	113.4	5.04	100.8	5.60	111.9
	5.23	104.5	5.01	100.2	4.82	96.4
	5.12	102.4	4.68	93.6	4.58	91.6
Average % recovery	5.34	106.8	5.18	103.5	5.65	113.0
Standard deviation	0.50		0.51		0.78	
Limit of Detection	1.49		1.51		2.33	

	Dinotefuran		Acetamiprid	
	[Sample] ng/L	% Recovery	[Sample] ng/L	% Recovery
	5.13	102.5	4.97	99.4
	5.40	108.0	5.66	113.2
	5.07	101.3	5.17	103.4
	5.57	111.5	5.86	117.2
	5.53	110.6	4.94	98.9
	5.63	112.7	5.41	108.2
	5.27	105.3	4.56	91.2
	5.16	103.2	4.20	83.9
Average % recovery	5.34	106.9	5.10	101.9
Standard deviation	0.22		0.55	
Limit of Detection	0.66		1.66	

S3. Calibration Summary

All target analytes are quantified using thiamethoxam-d3 as an internal standard. The coefficient of determination (r^2) values must be greater that 0.990 with initial calibration before continuation of the analytical run. Continuing calibration check standards (CCCS) are analyzed following 10 sample extract runs. A blank sample with internal standards is analyzed following 10 sample runs and the CCCS. A linear regression fit is used for quantification of all analytes except for imidacloprid using a 7- point series of standards ranging from 1.0 ng/ml to 100 ng/mL. A quadratic regression is used for imidacloprid using a 7-point series of standards ranging from 1.0 ng/ml to 100 ng/mL. The regression line is not forced through the origin without weighting of any standards.

Table S5. Recovery of Surrogate Standards*

Site	Aqueous % Recovery	POCIS % Recovery
Site 1 - Poncho	45.8	36.6
Site 2 - Tomorrow	47.2	36.2
Site 3 - L. Plover River	48.0	40.6
Site 4 - Isherwood Creek	40.3	33.9
Site 5 - 4-mile Creek	45.2	33.8
Site 5 – replicate	36.7	---
Site 6 - Bear Creek	55.5	29.2
Site7 - Emmons Creek	47.1	23.4
Site 8 - Pine Creek	40.8	26.9
Site 9 - White River	54.3	29.5
Site 10 Chaffee Creek	40.9	38.0
Site 11 - Tagatz Creek	57.2	46.9
Site 12 - Carter Creek	51.9	40.8
Site 12 – Carter Creek –rep.	---	28.3
Site 13 - Dry Creek	48.8	34.5
Site 14 - Big Roche-a-cri	37.0	37.5
Site 15 – Buckner Creek	46.6	45.2
Site 16 – Leola Ditch	53.2	35.6
Site 16 – Leola Ditch - rep	---	25.0
Site 17 - 10- mile Creek	47.2	35.9
Site 17 - 10-mile Creek - rep	---	36.5
Site 18 - 10-mile S. Branch	52.7	50.5
Site 19 - Ditch 5	48.0	41.8
Site 19- Ditch 5 replicate	47.8	41.8
Site 20 - 10-Mile N. Branch	54.4	51.0

*Aqueous samples and POCIS media were fortified with imidacloprid-d4 to assess recovery of neonicotinoids through the analytical process at 10 ng/L and 25 pg/ml respectively. Results are summarized on a per sample basis.

Table S6. Relative percent differences (RPD) of field deployed Polar Organic Compound Integrative Samplers (POCIS) in Wisconsin Central Sands streams

YEAR ONE⁑	Days deployed	Thiamethoxam (ng/L)	RPD	Clothianidin (ng/L)	RPD	Imidacloprid (ng/L)	RPD
Carter Creek	34	4.6	16%	9.9	18%	21.4	19%
Carter Creek field duplicate	34	4.0		8.3		17.7	
Ditch 5	29	205.4	2%	37.2	1%	29.9	5%
Ditch 5 field duplicate	29	208.9		37.7		31.5	
**YEAR TWO⁑**							
HWY 73	29	31.3	63%	6.9	46%	3.5	43%
HWY 73 duplicate	29	60.0		11.0		5.4	
HWP 13	32	26.7	2%	6.8	6%	3.4	5%
HWP 13 field duplicate	32	27.1		6.4		3.6	
CTY U	29	32.6	2%	5.0	18%	2.4	21%
CTY U field duplicate	29	33.4		6.0		2.9	
Evergreen	32	98.0	74%	4.6	27%	3.3	25%
Evergreen field duplicate	32	44.8		3.5		2.6	
Evergreen	31	30.1	81%	4.2	40%	2.6	36%
Evergreen field duplicate	31	71.3		6.4		3.7	
Townline	30	23.4	10%	12.6	11%	6.3	9%
Townline field duplicate	30	21.1		11.3		5.7	
Elm Rd.	31	65.2	43%	23.4	40%	9.9	40%
Elm Rd. field duplicate	31	41.9		15.5		6.6	
Taft Rd.	31	52.2	9%	46.9	20%	19.2	19%
Taft Rd. field duplicate	31	47.7		38.3		15.9	
Taft Rd.	28	36.3	31%	24.2	2%	8.2	10%
Taft Rd. field duplicate	28	49.6		23.8		7.4	
CTY D	32	29.1	55%	84.7	42%	27.4	55%
CTY D field duplicate	32	16.5		55.2		15.6	

⁑Year One accounts for the POCIS deployed during the study presented in this manuscript. Year Two included to allow the reader to see a greater number of replicate field deployed POCIS.

S4. Groundwater Flow Modeling

A groundwater flow model that had been developed for the Wisconsin Sand Plain was adapted for this project (Kraft et al., 2012). The model was created using the USGS MODFLOW program (Niswonger et al., 2011). The model described an area of approximately 9,000 km^2 in central Wisconsin. The modeled area was bounded by the Wisconsin River on the west and tributaries to the Fox and Wolf Rivers on the east. The model used 200 meter x 200 meter grid cells and two layers. The upper layer represented the permeable glacially outwash and tills. The lower layer represented the less permeable sandstone bedrock. The model uses a spatially variable hydraulic conductivity ranging from 4 to 60 meter/day with a mean of 29 meter/day within the groundwater contributing areas for the sampling sites. The recharge in the model was calibrated to the long-term average water table and streamflow conditions and ranged from 5.9 to 10.3 inches/year in the contributing areas for the sampling sites. That model underestimated the streamflow that was observed during our study period with a difference between measured and modeled flows that was largest (as a percent) in the headwater streams. That is consistent with the study occurring following several consecutive years of above average precipitation leading to a period of higher water table elevation. To account for the higher flow conditions during the study period and delineate contributing areas in the headwater sampling sites, we adjusted the recharge in the model to improve the agreement with the observed streamflow. A steady-state approximation as used with adjustments to the recharge rate. By adjusting the recharge to a constant 13 inches yr^{-1}, we were able to reasonably match the August streamflow at the sampling sites. This recharge rate is within the year-to-year range of 3 to 22 inches for this region (Weeks & Stangland, 1971). Figure S2 shows the agreement between the modeled and measured streamflow. The MODFLOW groundwater flow model results were used with the particle tracking model MODPATH to simulate groundwater flow patterns and times of travel in the aquifer (Pollock, 2016). We used

forward particle tracking and assumed a porosity of 0.3 that was between previous estimates of 0.15 for specific yield and 0.40 for volumetric water content (Fienen et al., 2021; Kraft & Stites, 2003). The particle tracking used uniformly distributed particles at 100 meter spacing and identified those starting locations that terminated in the stream upstream of the sampling locations. The particle travel times to the stream were used to divide the full groundwater contributing area into different travel time zones. Figure S3 shows the travel time age distribution simulated for the streamflow at each location. Those results estimate that one-third to two-thirds of the flow at each station is less than twenty years old. Groundwater flow and particle tracking models were used with FloPy (Bakker et al., 2025) and simulation results were processed using R (R Core Team, 2013).

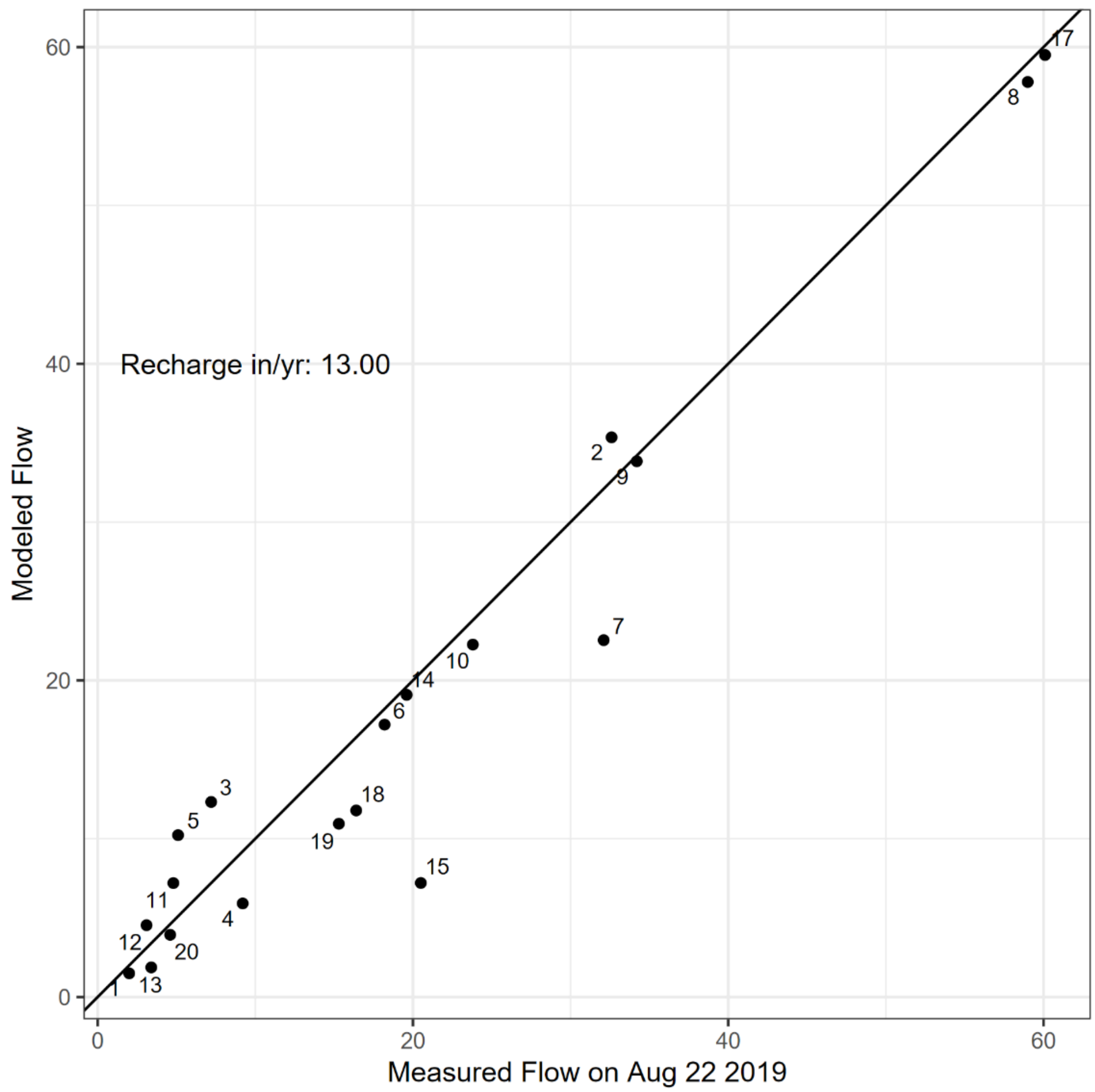

Figure S2. Agreement between flow measured on August 22 and the modeled flow assuming the steady-state recharge rate and size of the groundwater contributing areas.

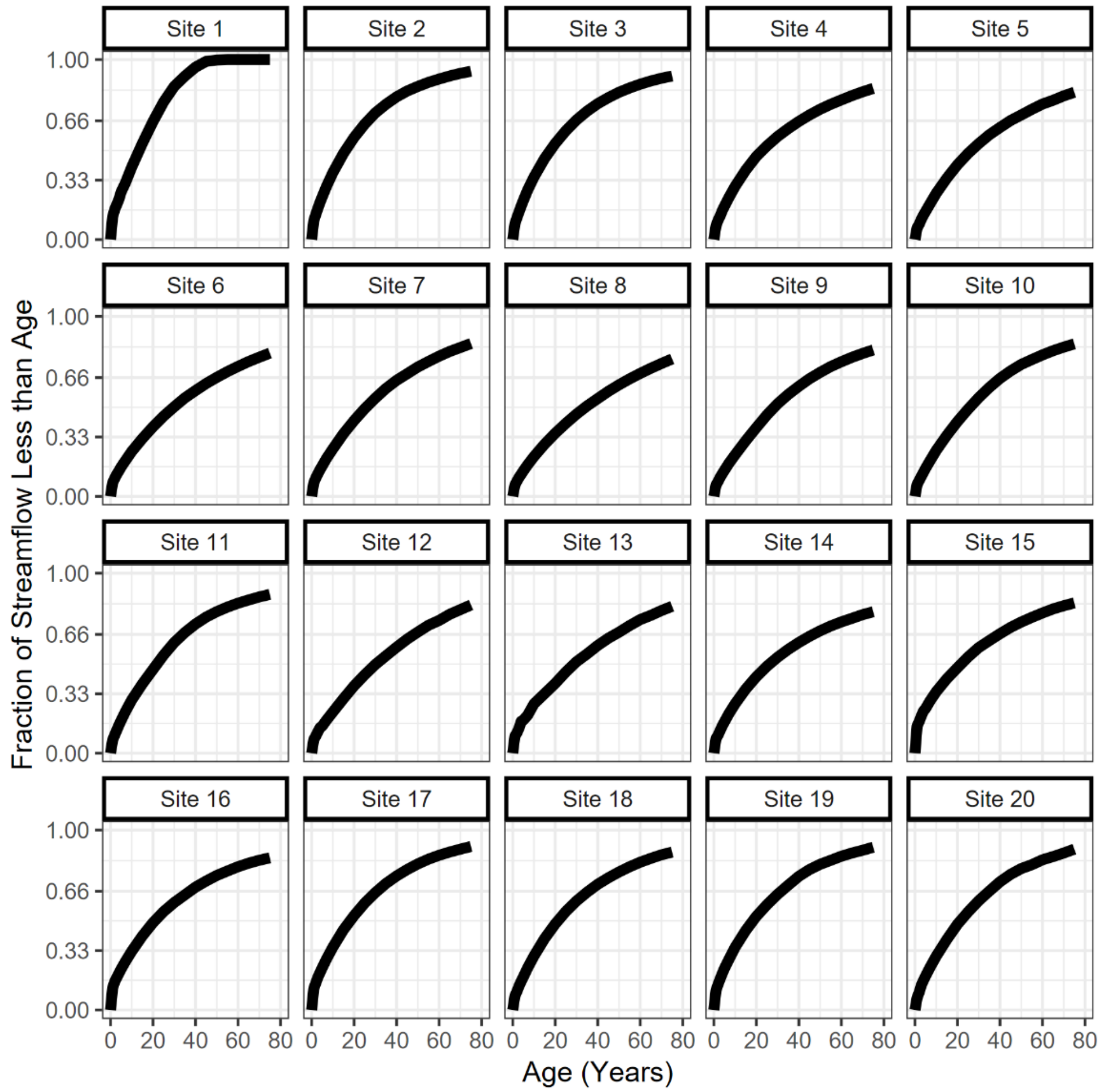

Figure S3. Groundwater travel time distributions for the streamflow at the sampling locations developed from the groundwater flow modeling.

S5. Chemical Characteristics of the Neonicotinoids

Chemical characteristics of the three neonicotinoids are shown below. The organic carbon-based partition coefficient, K_{OC}, was used to estimate an aquifer retardation factor, R_d, with aquifer properties estimated based on previous studies. The R_d of 1.1 to 1.2 implies compounds have low retention within the aquifer and would travel at 80%-90% of the groundwater velocity.

Table S7. Chemical characteristics of the neonicotinoids

Compound	Water Solubility (mg L^{-1})	K_{OC} (L Kg^{-1})	Aquifer R_d Estimated***
Imidacloprid	610*	140**	1.22
Thiamethoxam	4100*	56*	1.09
Clothianidin	327*	123*	1.20

*Water solubility and K_{OC} from the Pesticide Properties Database (PPDB) (Lewis et al., 2016) https://sitem.herts.ac.uk/aeru/ppdb/en/index.htm, accessed September 4, 2025

**Imidacloprid K_{OC} estimated from Zheng et al. (Zheng et al., 2021) although other literature values range from 60 to more than 500 (Flores-Céspedes et al., 2002; Uthman et al., 2024) and other studies report isotherm nonlinearity (e.g., Freundlich n = 0.7-0.9).

***R_d estimated using aquifer bulk density 1.6 g cm^{-3}, porosity of 0.3, fraction of organic carbon (foc) = 0.0002 (Stevenson et al., 2024; Yost et al., 2019) and calculated as 1 + (bulk density/porosity) * K_{OC} * f_{OC} (Fetter, 2001).

S6. Neonicotinoid Use History in the Wisconsin Sand Plain

Figure S4 shows neonicotinoid use estimated by the United States Geologic Survey (USGS) for three commonly used neonicotinoids in the agriculturally important Wisconsin (US) Central Sands region (Wieben, 2021). By 2015, approximately 80% of all NNI globally were used as seed coatings (Bass et al., 2015). The importance of seed treatment is evident in Figure S4 by the apparent reduction in estimated use of thiamethoxam and clothianidin after 2014 when seed coatings were no longer included in the estimates (Wieben, 2021).

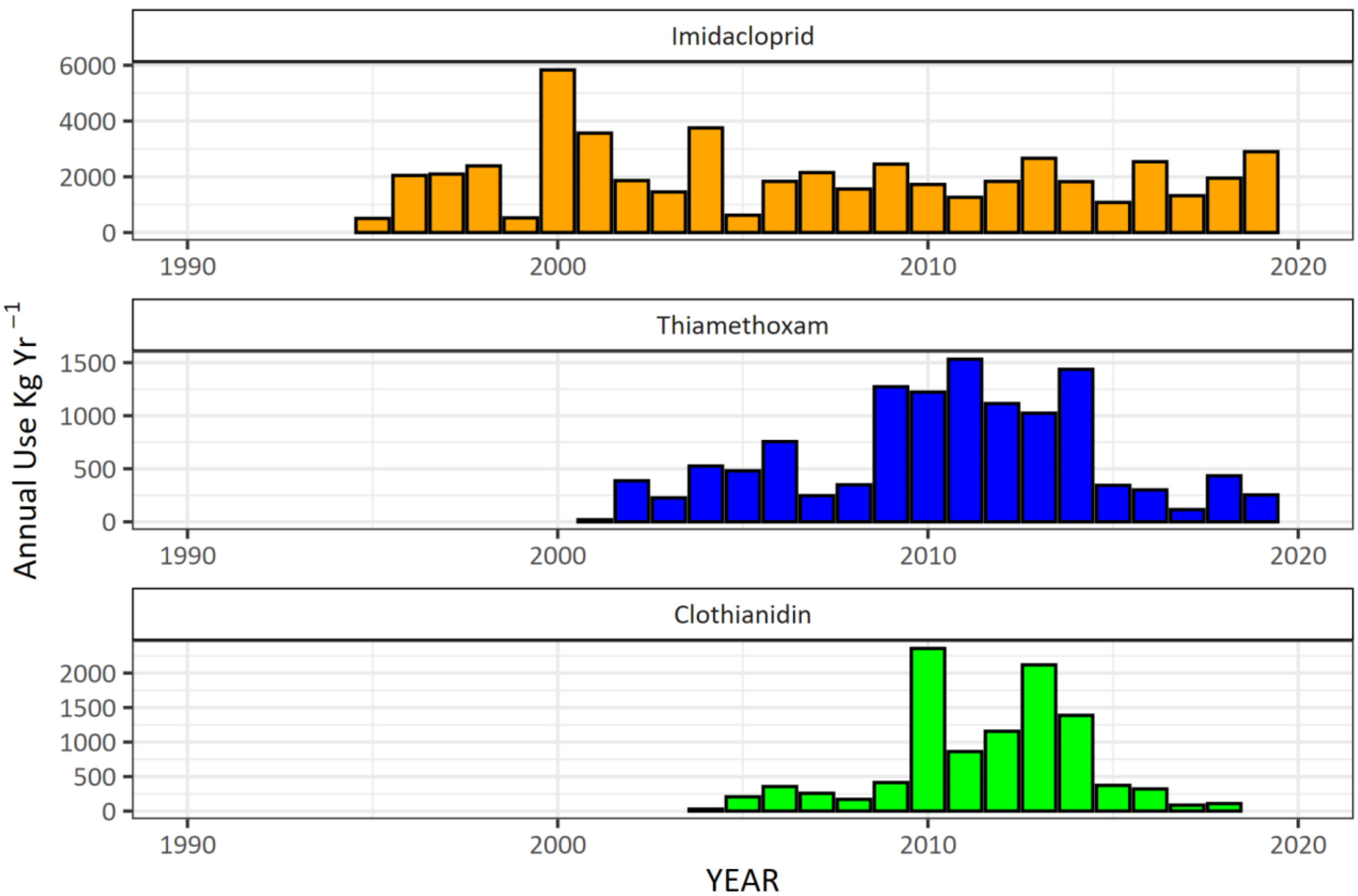

Figure S4. History of neonicotinoid use in central Wisconsin (Adams, Portage and Waushara Counties) shown as annual estimated usage (USGS EPest High (Wieben, 2021). The apparent reduction in use after 2014 includes a change in the estimation method to no longer include seed treatments in the total.

S7. Field-Edge Groundwater Monitoring Wells

The Wisconsin Department of Agriculture, Trade and Consumer Protection has been monitoring a set of field-edge monitoring well nests in the Wisconsin Central Sands since 2008. Well locations, construction and neonicotinoid monitoring from these wells has been described previously (Bradford et al., 2018; Huseth & Groves, 2014; Senger et al., 2019). These wells have 1.5 m (5 ft) or 3 m (10 ft) screens at different depths at the edge of potato/vegetable agricultural fields. The upper screened interval usually intersects the top of the water table. We are using results from only the upper 3 m of screened interval that were collected between 2008-2025 (Ken Potrykus, Wisconsin Department of Agriculture, Trade and Consumer Protection, personal communication).

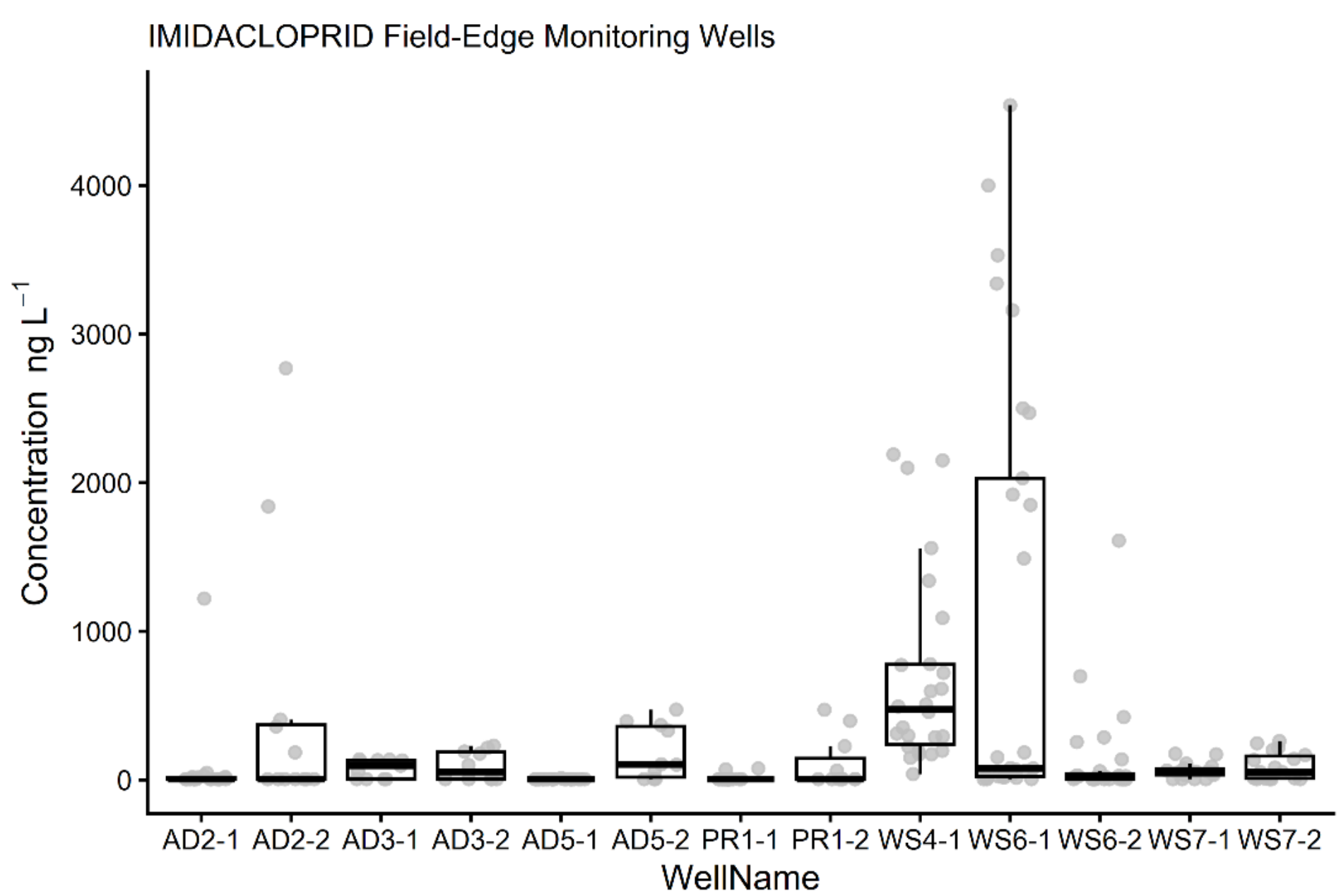

Figure S5. Imidacloprid concentrations in field-edge monitoring wells. Individual values as grey symbols, median as thick line, inner two quartiles as box.

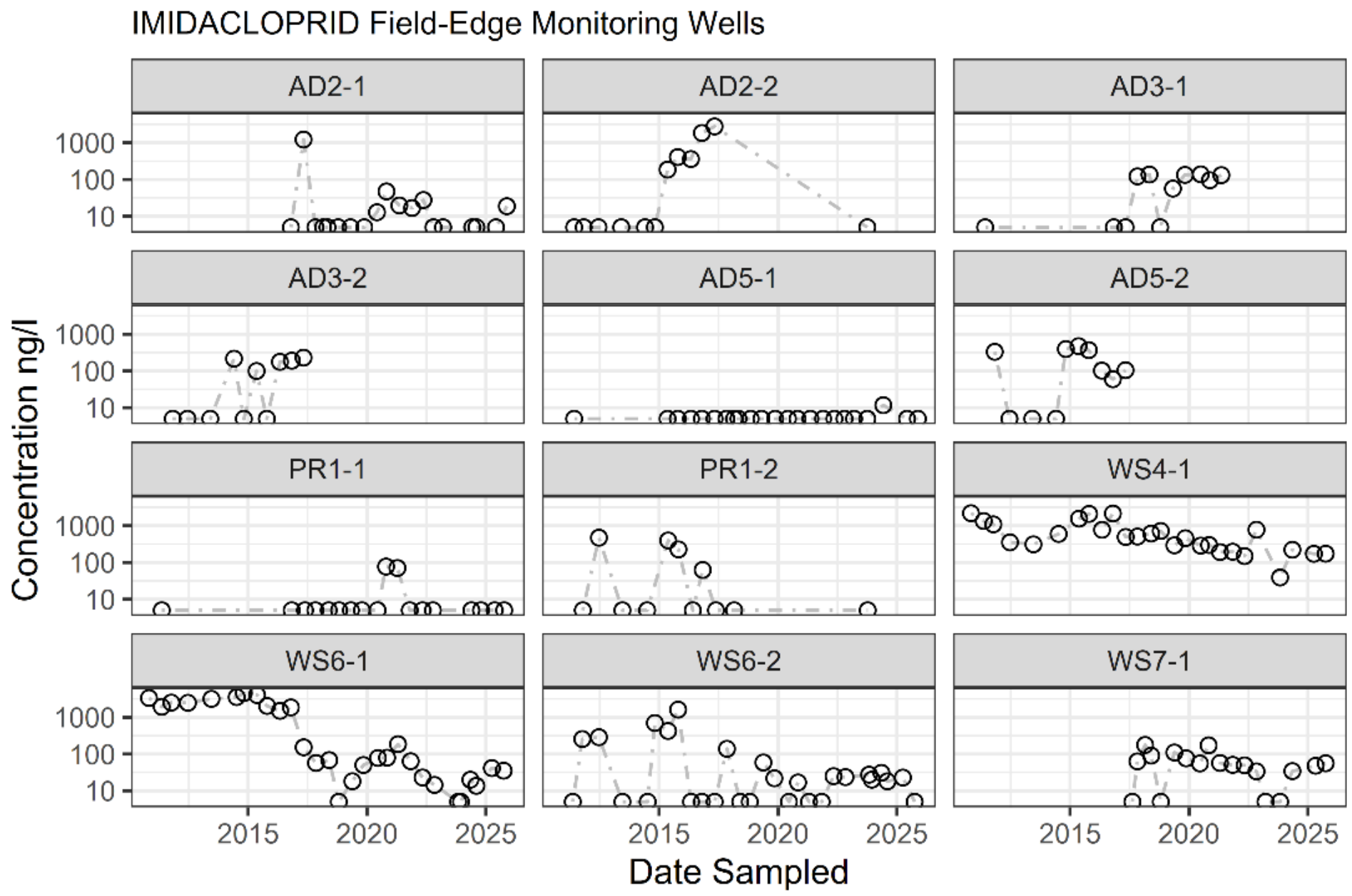

Figure S6. Imidacloprid concentrations in field-edge monitoring wells over time. Values below the reporting limit (10 ng L^{-1}) plotted as 5 ng L^{-1}.

Table S8. Imidacloprid Field-Edge Monitoring Well Summary (ng L^{-1})

Characteristic	AD2-1 N = 19	AD2-2 N = 12	AD3-1 N = 11	AD3-2 N = 10	AD5-1 N = 23	AD5-2 N = 10
Concentration						
Mean	75	466	76	94	5	185
SD	278	893	61	100	1	185
Median	5	5	95	52	5	104
Q1	5	5	5	5	5	5
Q3	19	383	133	191	5	369
Min	5	5	5	5	5	5
Max	1220	2770	138	230	12	473

Characteristic	PR1-1 N = 18	PR1-2 N = 11	WS4-1 N = 26	WS6-1 N = 29	WS6-2 N = 28	WS7-1 N = 18
Concentration						
Mean	13	109	695	1095	133	61
SD	22	175	650	1480	330	51
Median	5	5	476	78	19	53
Q1	5	5	222	23	5	34
Q3	5	227	779	2030	45	76
Min	5	5	39	5	5	5
Max	77	472	2190	4540	1610	176

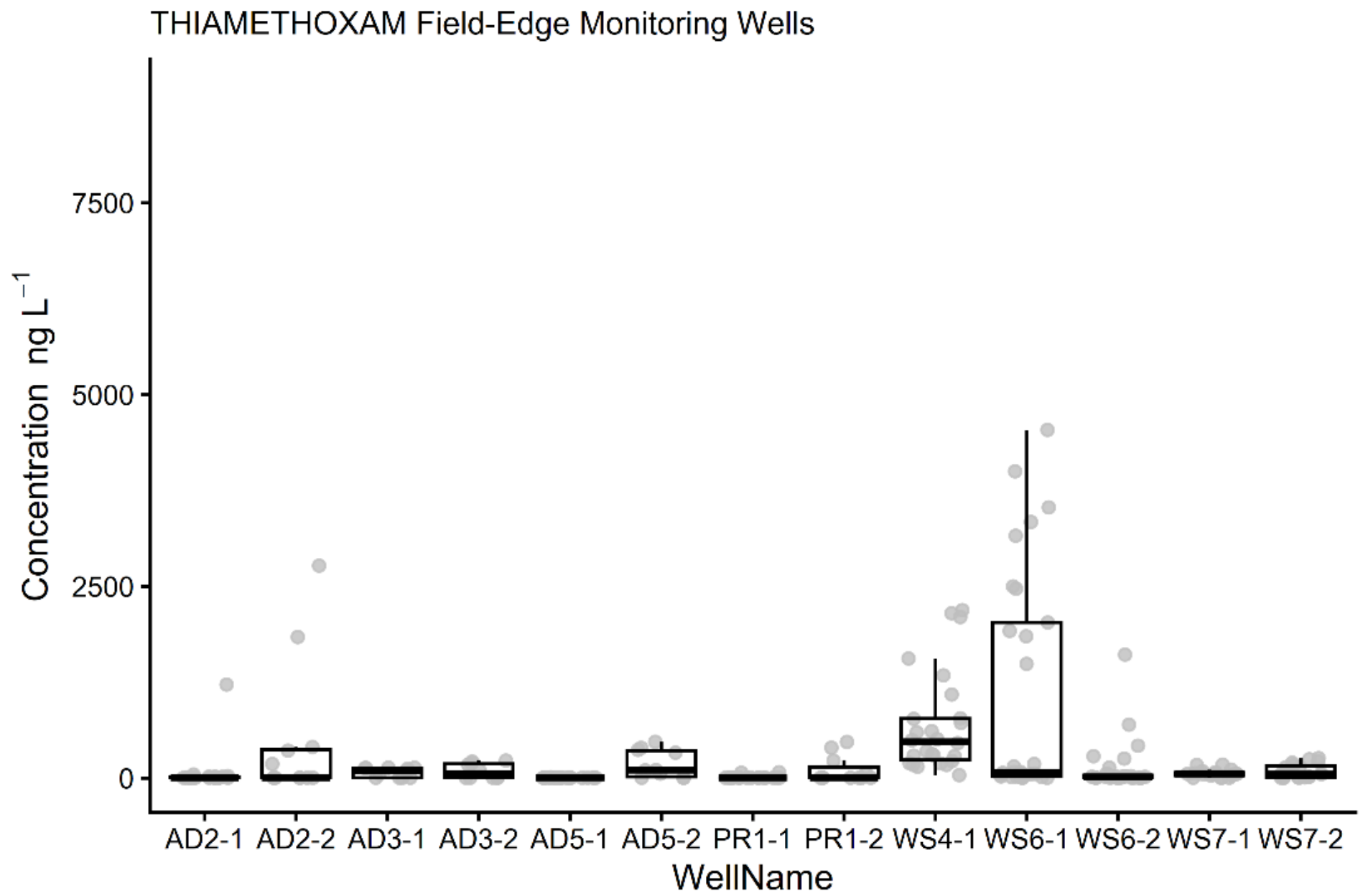

Figure S7. Thiamethoxam concentrations in field-edge monitoring wells. Individual values as grey symbols, median as thick line, inner two quartiles as box.

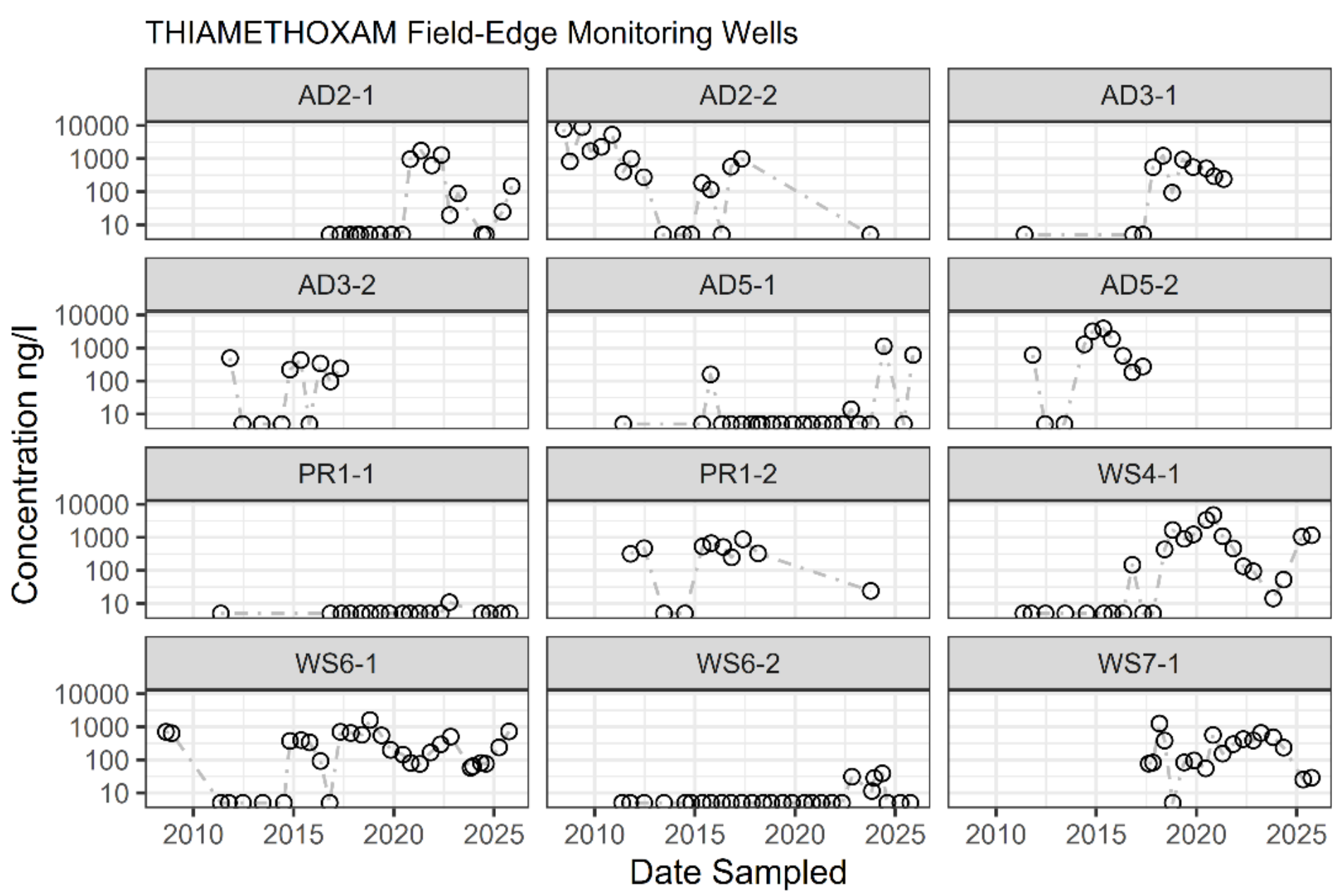

Figure S8. Thiamethoxam concentrations in field-edge monitoring wells over time. Values below the reporting limit (10 ng L^{-1}) plotted as 5 ng L^{-1}.

Table S9. Thiamethoxam Field-Edge Monitoring Well Summary (ng L^{-1})

Characteristic	AD2-1 N = 19	AD2-2 N = 18	AD3-1 N = 11	AD3-2 N = 10	AD5-1 N = 23	AD5-2 N = 10
Concentration						
Mean	260	1687	399	185	88	1191
SD	513	2756	401	190	262	1380
Median	5	486	291	159	5	599
Q1	5	5	5	5	5	184
Q3	146	1690	550	340	5	1880
Min	5	5	5	5	5	5
Max	1740	8930	1220	495	1130	3890

Characteristic	PR1-1 N = 18	PR1-2 N = 11	WS4-1 N = 25	WS6-1 N = 30	WS6-2 N = 28	WS7-1 N = 18
Concentration						
Mean	5	362	664	311	8	293
SD	1	283	1156	350	9	313
Median	5	328	94	183	5	194
Q1	5	24	5	64	5	77
Q3	5	530	1040	547	5	423
Min	5	5	5	5	5	5
Max	11	873	4740	1600	39	1250

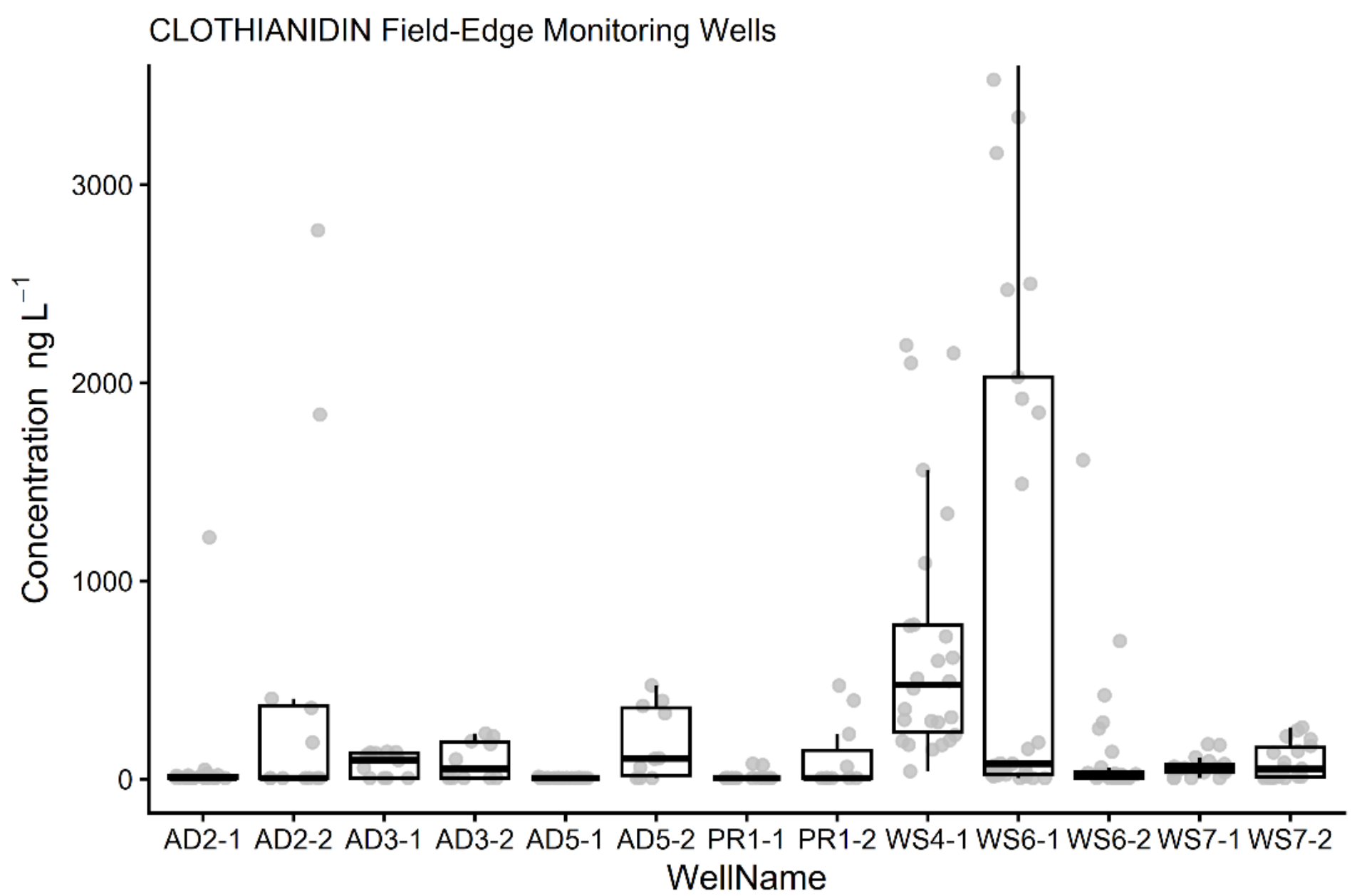

Figure S9. Clothianidin concentrations in field-edge monitoring wells. Individual values as grey symbols, median as thick line, inner two quartiles as box.

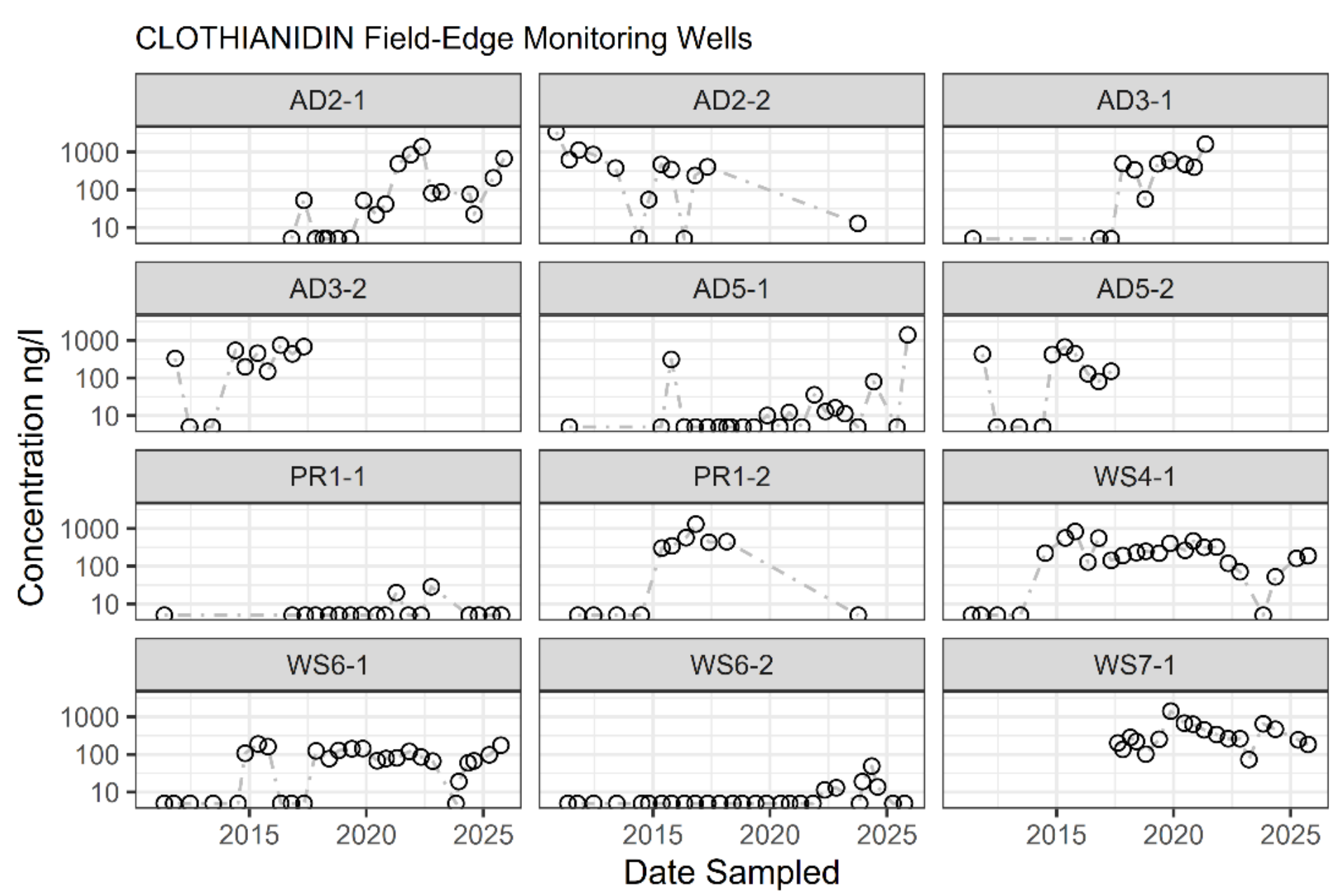

Figure S10. Clothianidin concentrations in field-edge monitoring wells over time. Values below the reporting limit (10 ng L^{-1}) plotted as 5 ng L^{-1}.

Table S10. Clothianidin Field-Edge Monitoring Well Summary (ng L^{-1})

Characteristic	AD2-1 N = 19	AD2-2 N = 13	AD3-1 N = 11	AD3-2 N = 10	AD5-1 N = 23	AD5-2 N = 10
Concentration						
Mean	214	613	409	354	85	233
SD	375	913	466	262	294	236
Median	52	377	397	382	5	140
Q1	5	55	5	150	5	5
Q3	207	628	497	541	13	430
Min	5	5	5	5	5	5
Max	1380	3430	1630	743	1400	662

Characteristic	PR1-1 N = 18	PR1-2 N = 11	WS4-1 N = 25	WS6-1 N = 28	WS6-2 N = 28	WS7-1 N = 18
Concentration						
Mean	7	312	229	73	8	380
SD	6	396	208	60	9	316
Median	5	303	192	73	5	262
Q1	5	5	71	5	5	201
Q3	5	448	317	122	5	465
Min	5	5	5	5	5	73
Max	28	1310	835	189	49	1410

References

Bakker, M., Post, V. H., Hughes, J. D., Langevin, C. D., White, J. T., Leaf, A. T.,…Bonelli, W. P. (2025). *FloPy*. In *U.S. Geological Survey Software Release* (Version 3.10.0.dev3) https://doi.org/10.5066/F7BK19FH

Bass, C., Denholm, I., Williamson, M. S., & Nauen, R. (2015). The global status of insect resistance to neonicotinoid insecticides. *Pesticide Biochemistry and Physiology*, *121*, 78-87. https://doi.org/10.1016/j.pestbp.2015.04.004

Bradford, B. Z., Huseth, A. S., & Groves, R. L. (2018). Widespread detections of neonicotinoid contaminants in central Wisconsin groundwater. *PLOS One*, *13*(10), e0201753. https://doi.org/10.1371/journal.pone.0201753

Fetter, C. W. (2001). *Applied Hydrogeology* (4th ed.). Prentice Hall.

Fienen, M. N., Haserodt, M. J., Leaf, A. T., & Westenbroek, S. M. (2021). *Appendix C: Central Sands Lakes Study Technical Report: Modeling Documentation* (Central Sands Lake Study Report Prepared for the Wisconsin Legislature, Issue. Wisconsin Department of Natural Resources. https://dnr.wisconsin.gov/topic/Wells/HighCap/CSLStudy.html

Flores-Céspedes, F., González-Pradas, E., Fernández-Pérez, M., Villafranca-Sánchez, M., Socías-Viciana, M., & Ureña-Amate, M. D. (2002). Effects of Dissolved Organic Carbon on Sorption and Mobility of Imidacloprid in Soil. *Journal of Environmental Quality*, *31*(3), 880-888. https://doi.org/https://doi.org/10.2134/jeq2002.8800

Huseth, A. S., & Groves, R. L. (2014). Environmental Fate of Soil Applied Neonicotinoid Insecticides in an Irrigated Potato Agroecosystem. *PLOS One*, *9*(5), Article e97081. https://doi.org/10.1371/journal.pone.0097081

Kraft, G. J., Mechenich, D. J., & Haucke, J. (2012). *Information Support for Groundwater Management in the Wisconsin Central Sands, 2009-2011* (A Report to the Wisconsin Department of Natural Resources in Completion of Project NMA00000253, https://www3.uwsp.edu/cnr-ap/watershed/Documents/kraft_centralsands_2012.pdf

Kraft, G. J., & Stites, W. (2003). Nitrate impacts on groundwater from irrigated-vegetable systems in a humid north-central US sand plain. *Agriculture, Ecosystems & Environment*, *100*(1), 63-74. https://doi.org/https://doi.org/10.1016/S0167-8809(03)00172-5

Lewis, K. A., Tzilivakis, J., Warner, D. J., & Green, A. (2016). An international database for pesticide risk assessments and management. *Human and Ecological Risk Assessment: An International Journal*, *22*(4), 1050-1064. https://doi.org/10.1080/10807039.2015.1133242

Niswonger, R. G., Panday, S., & Ibaraki, M. (2011). *MODFLOW-NWT, a Newton formulation for MODFLOW-2005* U.S. Geological Survey Techniques and Methods 6-A37, 44 p., https://doi.org/10.3133/tm6A37.

Pollock, D. W. (2016). *User guide for MODPATH version 7-A : a particle tracking model for MODFLOW*. U.S. Department of the Interior, U.S. Geological Survey.

R Core Team, R. (2013). R: A language and environment for statistical computing.

Senger, S., Blanchard, D., Cook, C., Debaker, A., Bowman, L., Kelley, G. (2019). Neonicotinoid pesticides in Wisconsin groundwater and surface water. In: Wisconsin Department of Agriculture, Trade and Consumer Protection: Madison, WI.

Stevenson, A., Zhang, Y., Huang, J., Hu, J., Paustian, K., & Hartemink, A. E. (2024). Rates of soil organic carbon change in cultivated and afforested sandy soils. *Agriculture, Ecosystems & Environment*, *360*, 108785. https://doi.org/https://doi.org/10.1016/j.agee.2023.108785

Uthman, Q. O., Kadyampakeni, D. M., Leiva, J. A., Judy, J. D., & Nkedi-Kizza, P. (2024). Sorption and degradation processes of imidacloprid in Florida soils. *PLOS One*, *19*(9), e0305006. https://doi.org/10.1371/journal.pone.0305006

Weeks, E. P., & Stangland, H. G. (1971). *Effects of irrigation on streamflow in the Central Sand Plain of Wisconsin.* US Geological Survey Open-File Report 70-362.

Wieben, C. (2021). Estimated annual agricultural pesticide use by major crop or crop group for states of the conterminous United States, 1992–2019 (including preliminary estimates for 2018–19). 2021. *US Geological Survey data release*.

Yost, J. L., Roden, E. E., & Hartemink, A. E. (2019). Geochemical Fingerprint and Soil Carbon of Sandy Alfisols. *Soil Systems*, *3*(3), Article 59. https://doi.org/10.3390/soilsystems3030059

Zheng, T., Hu, T., Zhang, J., Tang, C., Duan, J., Song, Y., & Zhang, Q. (2021). Dynamics in imidacloprid sorption related to changes of soil organic matter content and quality along a 20-year cultivation chronosequence of citrus orchards. *Environmental Pollution*, *291*, 118069. https://doi.org/https://doi.org/10.1016/j.envpol.2021.118069
